# Matrix Metalloproteinase-9 (*MMP-9*) polymorphisms in patients with cutaneous malignant melanoma

**DOI:** 10.1186/1471-2350-8-10

**Published:** 2007-03-08

**Authors:** Javier Cotignola, Boris Reva, Nandita Mitra, Nicole Ishill, Shaokun Chuai, Ami Patel, Shivang Shah, Gretchen Vanderbeek, Daniel Coit, Klaus Busam, Allan Halpern, Alan Houghton, Chris Sander, Marianne Berwick, Irene Orlow

**Affiliations:** 1 Memorial Sloan-Kettering Cancer Center, New York, NY, USA; 2 University of Pennsylvania, Philadelphia, PA, USA; 3 University of New Mexico, Albuquerque, NM, USA

## Abstract

**Background:**

Cutaneous Malignant Melanoma causes over 75% of skin cancer-related deaths, and it is clear that many factors may contribute to the outcome. Matrix Metalloproteinases (MMPs) play an important role in the degradation and remodeling of the extracellular matrix and basement membrane that, in turn, modulate cell division, migration and angiogenesis. Some polymorphisms are known to influence gene expression, protein activity, stability, and interactions, and they were shown to be associated with certain tumor phenotypes and cancer risk.

**Methods:**

We tested seven polymorphisms within the *MMP-9 *gene in 1002 patients with melanoma in order to evaluate germline genetic variants and their association with progression and known risk factors of melanoma. The polymorphisms were selected based on previously published reports and their known or potential functional relevance using *in-silico *methods. Germline DNA was then genotyped using pyrosequencing, melting temperature profiles, heteroduplex analysis, and fragment size analysis.

**Results:**

We found that reference alleles were present in higher frequency in patients who tend to sunburn, have family history of melanoma, higher melanoma stage, intransit metastasis and desmoplastic melanomas among others. However, after adjustment for age, sex, phenotypic index, moles, and freckles only Q279R, P574R and R668Q had significant associations with intransit metastasis, propensity to tan/sunburn and primary melanoma site.

**Conclusion:**

This study does not provide strong evidence for further investigation into the role of the *MMP-9 *SNPs in melanoma progression.

## Background

Cutaneous Malignant Melanoma (CMM) causes over 75% of skin cancer-related deaths. Melanoma incidence has been rising steadily, with 62,190 new cases and 7,910 deaths estimated for 2006 in the United States [1]. It is becoming clear that genetic factors contribute to the development and/or progression of the disease, as well as the exposure to ultraviolet light (UV) and tumor microenvironment.

Invasive primary melanomas are aggressive and prone to metastasis. Proteolytic degradation of the extracellular matrix (ECM) constituents may release and activate ECM-bound cytokines and ECM fragments (matrikines) that modulate cell growth, migration and angiogenesis [2,3]. Under physiological conditions, matrix metalloproteinase (MMP) expression is low in most cells; however, many tumor types show a dramatically overexpression of these enzymes. This, in turn, produces an overactive proteolysis of the ECM and basement membrane, promoting cancer invasion through a variety of biochemical, biophysical, and signal transduction mechanisms.

Liu *et al *found that the MMP-2 and MMP-9 protein levels were higher in breast tumor tissues when compared to the corresponding normal tissue (p < 0.01) and that MMP-2 was significantly increased in larger tumors (p < 0.0001), and in metastatic lesions (p < 0.05) [4]. MMP-9 expression was significantly higher in colorectal tumors when compared to the adjacent normal tissue (p < 0.05) [5]. In melanoma, there is some evidence suggesting a role of MMPs in the progression of the disease. It has been demonstrated that invasive melanoma cell lines show higher MMP-9 expression and higher activity when compared to non-invasive cell lines [6,7]. In primary melanomas, MMP-9 is variably expressed in radial but not in the vertical growth phase and the *de novo *expression seems associated with early invasion [8]. MMP-2 was evident by immunohistochemistry in malignant melanoma lesions, but not in benign and atypical nevi [9]. Corte *et al *found an association between MMP-13 expression with mitotic index (p = 0.002) in CMM [10]. Circulating blood levels of MMP-9 may be useful in predicting progression in patients with melanoma although it has been suggested that serum levels contain higher levels of proteases released during the clotting process, and therefore, the results obtained by Nikkola *et al *should be validated using plasma samples [11-14].

Some polymorphisms are known to influence gene expression, protein activity, protein stability and protein-protein interactions, and some of them are associated with an increased risk for cancer and other diseases. To date, and to the best of our knowledge, there are no reports on associations between polymorphisms in *MMP-9 *and melanoma.

Polymorphisms in the promoter of MMP-9 have been implicated in the regulation of gene expression and susceptibility to various diseases. Two out of five identified sequence variants are functionally relevant: a single nucleotide polymorphism at (-)1562 bp (C → T) and a (CA)_n _microsatellite at position (-)131 bp. The C to T substitution at position (-)1562 results in a loss of binding of a nuclear protein and an increase in transcriptional activity in macrophages [15]. Similarly, the highest promoter activity has been observed in reporter constructs containing 21 or 23 (CA) tandem repeats, suggesting that the number of repeats modulates the transcriptional activity [15].

To test the hypothesis that polymorphisms in the *MMP-9 *gene [GenBank: AF538844, OMIM: 120361] may influence melanoma progression, we examined seven different polymorphisms in a cohort of 1002 patients with melanoma in order to evaluate germline genetic variants in the *MMP-9 *gene and their association with progression and known risk factors of melanoma.

## Methods

### Study population

We recruited 1002 patients with melanoma (stages 0–IV) at Memorial Sloan-Kettering Cancer Center (MSKCC), New York, USA. The study protocol was approved by the MSKCC Institutional Review Board (IRB). Ninety-six percent of the patients approached signed an informed consent and agreed to participate in the study. Patients filled out a short self-administered questionnaire that included information on gender, race, age, family history, freckling density, hair and eye color, propensity to burn, and ability to tan after sun exposure. The information on hair color, eye color, and propensity to tan or sunburn were combined into a single variable, the "phenotypic index" [16]. This index, with minimum and maximum values of 1 and 5, represents the sum of points assigned to the following phenotypic features: hair color (1 if brown/black; 2 if light brown/blond; 3 if red/auburn); eye color (0 if brown; 1 if green/hazel/blue); and propensity to tan or sunburn (0 if tend to tan; 1 if tend to sunburn). We also obtained clinicopathological information including presence of dysplastic nevi, multiple primary tumors, stage at diagnosis and at follow-up (based on the AJCC 2002 classification), disease status, disease progression, and survival among others. The characteristics of the study group are shown in Table 1.

**Table 1:** Clinico-pathological characteristics of the study group

**Variable**	**Patients**	**%**
***Gender***		
Males	573	57.2
Females	429	42.8
***Family History***		
Yes	167	16.7
No	826	82.4
Unknown	9	0.9
***Multi-primary Melanoma***		
Yes	152	15.2
No	849	84.7
Unknown	1	0.1
***Stage at Diagnosis***		
0	58	5.8
I	504	50.3
II	235	23.4
III	169	16.9
IV	9	0.9
Unstagable^¥^	27	2.7
***Current Stage***		
0	56	5.6
I	435	43.4
II	159	15.9
III	217	21.7
IV	129	12.9
Unstagable^¥^	6	0.5
***Primary Clark Level***		
I	58	5.8
II	108	10.8
III	150	14.9
IV	489	48.8
V	69	6.9
Unknown	128	12.8
***Thickness (mm)***		
In situ	58	5.8
< 1.01	321	32.0
1.01 – 2.00	272	27.1
2.01 – 4.00	161	16.1
> 4.00	121	12.1
Unknown	69	6.9
***Distant Metastasis***		
Yes	130	13.0
No	870	86.8
N/A^φ^	2	0.2
***Phenotypic Index***		
1 (low risk)	38	3.8
2	210	21.0
3	317	31.6
4	328	32.7
5 (high risk)	105	10.5
N/A^φ^	4	0.4
***Site of the Primary Melanoma***		
Extremities	535	53.4
Trunk	342	34.1
Head and Neck	71	7.1
Non-cutaneous^ω^	12	1.2
Unknown	42	4.2

^¥^due to missing data on the "T" classification

^φ^N/A: not available

^ω^90% mucosal melanomas and 10% other sites.

### Biospecimens

Blood was obtained from 17 patients and buccal cells were collected from 985 individuals. DNA from buccal cells was extracted using Puregene^® ^kits (Gentra Systems Inc., Minneapolis, USA), and DNA from blood was extracted with the QIAamp DNA Blood kit (QIAGEN Inc. Valencia, USA) using manufacturer's recommendations. DNA concentration was measured by spectrophotometry at 260 nm in a Spectramax Plus 384 (Molecular Devices, Sunnyvale, USA). The DNA quality was determined by the ratio A260/A280.

### Selection of SNPs

All known non-synonymous variations in *MMP-9 *available in the dbSNP database of the National Center for Biotechnology Information (NCBI) [17] were evaluated with *in-silico *methods to determine which ones potentially disturb the molecular structure and/or function of MMP-9. In the analysis we determined: 1) the steric distortions and hydrogen bond losses caused by the polymorphisms; 2) the effect of the residue changes on the interactions with known ligands (metal cofactors, synthetic and natural inhibitors); and 3) the effect on conserved or specificity residues of four superfamilies of MMP-9 domains (MMP N-terminal domain, catalytic domain, fibronectin type II domain, hemopexin domain) by multiple sequence analysis [18].

### Genotyping

All genotyping was done with PCR-based methods and included: melting temperature analysis [19] coupled to the LightTyper instrument (Roche Applied Science, Indianapolis, USA), pyrosequencing [20] with the PSQ™ 96 MA or PSQ™HS 96A instruments (Biotage AB, Uppsala, Sweden), fragment size analysis [21] by an ABI PRISM^® ^3100 Genetic Analyzer (Applied Biosystems, Foster City, USA), and heteroduplex analysis [22,23] using the Wave DNA Fragment Analysis System (Transgenomic, Omaha, USA). The PCR primers and PCR conditions are listed in the Additional file 1. For the heteroduplex analysis, each sample was run unmixed and mixed with equimolar amount of wild type control. The *MMP-9 *(-)1562 C/T was assessed with a nested PCR because the largest fragment, although specific, was not suitable for pyrosequencing analysis. All genotyping included known internal controls and were considered for the analysis when there was 100% agreement between 2 independent laboratory members. Samples that failed were repeated once or twice, as needed.

### Sequencing

Samples were PCR amplified and then purified with a purification kit following the manufacturer's recommendations (QIAGEN Inc., Valencia, USA). One to 10 ng of each purified sample were sequenced in the DNA Sequencing Core Facility at Memorial Sloan-Kettering Cancer Center. Samples were run in an ABI 3730-XLDNA Analyzer (Applied Biosystems, Foster City, USA). Sequencing electropherograms were read at least twice, reviewed manually and with the Mutation Surveyor software, version 2.41 (SoftGenetics LLC, State College, USA).

### Statistical analysis

Two-sided Chi-Square tests, Cochran-Armitage tests for trend [24], and Fisher's exact tests were performed at each individual SNP in the *MMP-9 *gene to test for association with various clinical and epidemiologic factors. Multivariate analyses were conducted using logistic regression, adjusting for age, sex, phenotypic index, moles and freckles. To investigate associations between SNP and overall survival, time was measured from initial date of diagnosis with melanoma to date of death or last follow-up. Potential associations between time to recurrence, defined as a patient's first recurrence of melanoma, time to disease progression, and genotype were also examined. Survival estimates were computed using the methods of Kaplan and Meier [25] and comparisons between genotypes were made using the log-rank test. All statistical analyses were carried out using SAS version 9.1 (SAS Institute, Cary, NC). The EM algorithm [26] was used to estimate the haplotype frequencies and corresponding standard errors and confidence intervals were estimated using a jackknife process. For dichotomous traits, likelihood ratio tests were conducted to test for haplotype-trait association [27]. For continuous traits, a generalized linear models framework was utilized and score tests were constructed [28]. Omnibus test statistics over all haplotypes in addition to tests for association between each individual haplotype and the trait were performed. Exact p-values were computed using 1000 permutations. Analyses were conducted using SAS Genetics 9.1 software (SAS Institute Inc, Cary, North Carolina) and haplo.stat in R version 2.1.1 (The R Foundation for Statistical Computing, Vienna, Austria).

## Results

We included 1002 patients with melanoma stages 0 (*in situ*) to IV in the study. Ninety-five percent of these were cutaneous malignant melanoma patients; the rest included mucosal melanomas, other non-cutaneous sites, or unknown primary sites. Ninety-six percent were Caucasians followed by Hispanic (1.1%), black non-Hispanic (1.1%), and Asian/Indian (0.3%); fifteen had missing information on ethnicity and one refused to answer the question about race (1.5%). The age at diagnosis ranged from 5 to 89 years old (mean = 54 and median = 55).

### Evaluation of non-synonymous SNPs

There are 10 non-synonymous *MMP-9 *variations in the dbSNP (build 123). These variations are located in the activation peptide domain (A20V, E82K), catalytic domain (N127K, D165N, L447P, L449P), fibronectin type II domain (R279Q), and hemopexin domain (P574R, R621K, R668Q). Currently, there is no complete 3D structure of MMP-9 in any known biologically important state (precursor, active, bound to TIMP inhibitor). However, there are 3D structures of different fragments of MMP-9 and its homologs, including the complexes with TIMPs.

**Figure 1:**
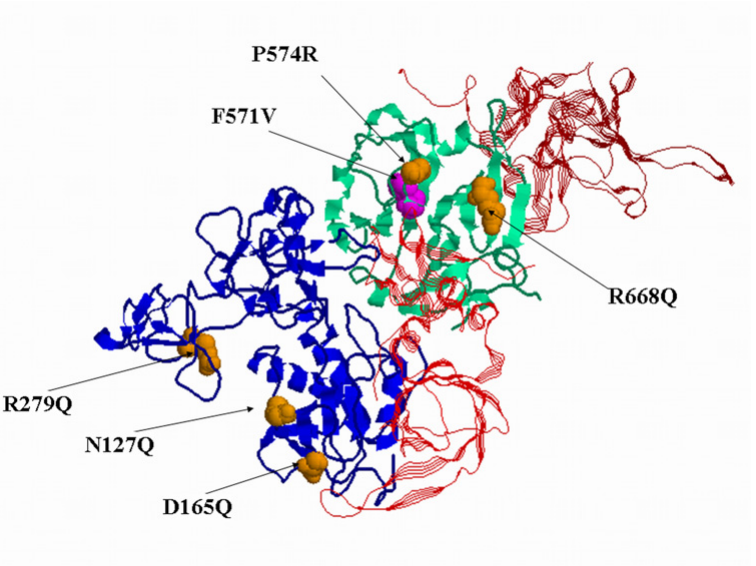
**Localization of studied SNPs on a model structure of MMP9**. The MMP-9 variations studied -N127K, D165N, Q279R, P574R, R668Q- are shown in context of MMP-9 3D structure. The reference residues have significant interactions with surrounding residues; therefore, it is likely that variations in these amino acids might disturb the protein stability and function. Residues N127, D165 are located in the catalytic domain (blue), Q279 is in the fibronectin type II domain (blue), and P574 and R668 are located in the hemopexin domain (green). The newly discovered variation F571V is shown in magenta. TIMP molecules, bound to the catalytic domain, are also depicted in the figure (red).

Therefore, we derived a synthetic structure of MMP-9 by 3D superposition of different structural fragments of MMP-9 and its close homolog MMP-2. Although this synthetic structure was not optimized for residue-residue contacts, it showed the MMP-9/TIMP binding site. Structure-based analysis of residue-residue interactions and analysis of protein domain family alignments suggested that five of the 10 non-synonymous SNPs -N127K, D165N, R279Q, P574R, and R668Q- would be appropriate candidates that might affect MMP-9 function and/or interaction with its inhibitors. None of the 5 polymorphic residues studied localizes to the MMP-9/TIMP binding interface (Figure 1), however all of these residues have specific interactions between their side-chain and surrounding residues, which are abolished as a result of an amino acid change. Our analysis is consistent with the results obtained by PolyPhen (Polymorphism Phenotyping [29]).

### Genotyping and statistical analysis

We genotyped one microsatellite and one SNP in the promoter of *MMP-9 *and five coding SNPs. The genotype and allele distributions are summarized in Tables 2 and 3, and were similar to those described in the dbSNP for European panels (HapMap-CEU, AFD EUR and PGA-European panels from dbSNP build 125) and previous published data. Hardy-Weinberg equilibrium was observed for all the polymorphisms.

**Table 2:** Frequency of the different SNP genotypes and alleles in the population studied

**Minor Allele**	n*	**Polymorphism**	**Reference ID**	Frequency (n*)	**Allele Frequency**
< 5%	538	N127K (C > G)	rs3918252	NN: 1.000 (538)	N: 1.000
				NK: 0.000 (0)	
				KK: 0.000 (0)	K: 0.000
				N/A: -- (0)	
		
		D165N (G > A)	rs8125581	DD: 1.000 (537)	D: 1.000
				DN: 0.000 (0)	
				NN: 0.000 (0)	N: 0.000
				N/A: -- (1)	
		
		P574R (C > G)	rs2250889	PP: 0.910 (485)	P: 0.954
				PR: 0.088 (47)	
				RR: 0.002 (1)	R: 0.046
				N/A: -- (5)	

> 5%	1002	(-)1562 C > T	rs3918242	CC: 0.721 (716)	C: 0.852
				CT: 0.262 (260)	
				TT: 0.017 (17)	T: 0.148
				N/A: -- (17)	
		
		Q279R (A > G)	rs2664538	QQ: 0.420 (420)	Q: 0.645
				QR: 0.451 (451)	
				RR: 0.130 (130)	R: 0.355
				N/A: -- (1)	
		
		R668Q (G > A)	rs2274756	RR: 0.718 (716)	R: 0.850
				RQ: 0.263 (262)	
				QQ: 0.019 (19)	Q: 0.150
				N/A: -- (5)	

*Total number of patients genotyped; N/A: not available due to genotyping failures

**Table 3:** Microsatellite (-) 131 (CA)_n _allele frequencies

**Number of (CA) repeats**	**Frequency (n)**
12	0.001 (1)
13	0.000 (0)
14	0.539 (1040)
15	0.017 (32)
16	0.001 (2)
17	0.001 (2)
18	0.000 (0)
19	0.018 (34)
20	0.017 (32)
21	0.169 (325)
22	0.140 (270)
23	0.084 (162)
24	0.015 (28)
N/A	-- (76)

N/A: not available due to genotyping failures

Statistically significant differences (p < 0.05) in genotype frequencies were observed in eleven different melanoma phenotypic groups. However, when we adjusted for age, sex, phenotypic index, moles, and freckles only four associations remained significant. The complete set of results is available as additional data (see Additional files 2, 3 and 4).

#### Promoter (-)131 (CA)_n_

The distribution of alleles for the microsatellite showed a bimodal distribution with a first high-frequency peak at 14 repeats (0.519) and a second low-frequency group of peaks with 21 and higher number of repeats (0.162, 0.135, and 0.081 for 21, 22, and 23 repeats respectively). Published data are contradictory with regard to the correlation between different promoter activity and different promoter lengths [30-32]. Therefore, we conducted the statistical analysis in two different ways. In the first analysis, we grouped the samples according to the presence of (CA)_14 _and in the second according to the presence of (CA)_≥ 21 _alleles (1 or 2 alleles *vs *none for both cases). No significant associations were found after adjustment for age, sex, phenotypic index, moles and freckles.

#### Promoter (-)1562 C/T

This polymorphism did not show any significant association with melanoma.

#### N127K and D165N

These polymorphisms showed no variation in our study group.

#### Q279R

The QQ genotype appeared more frequently in patients with intransit metastasis (p = 0.03, p_adjusted _= 0.02; Table 4). The same genotype was also more frequent among patients who tend to sunburn (p < 0.01, p_adjusted_< 0.01; Table 4).

**Table 4:** Statistically significant associations between the different SNPs and clinico-pathological variables

	**Genotype**		**p-value**
**Q279R**	QQ *(reference)*	QR + RR	

***Intransit Metastasis***			
Yes	17 (63%)	10 (37%)	
No	384 (41%)	557 (59%)	p = 0.03; p = 0.02^¥^
***Tan/Burn Tendency***			
Tend to Tan	26 (29%)	65 (71%)	
Tend to Sunburn	394 (43%)	516 (57%)	p < 0.01; p < 0.01^¥^

**P574R**	PP *(reference)*	PR + RR	

***Site***			
Extremities	263 (88%)	36 (12%)	
Trunk	164 (96%)	7 (4%)	
Head & Neck	36 (97%)	1 (3%)	
Non-cutaneous	6 (100%)	0 (0%)	p = 0.01; p = 0.02^¥^

**R668Q**	RR *(reference)*	RQ + QQ	

***Tan/Burn Tendency***			
Tend to Tan	55 (60%)	36 (40%)	
Tend to Sunburn	661 (73%)	245 (27%)	p = 0.01; p = 0.02^¥^

^**¥ **^p-value adjusted for: age, sex, phenotypic index, moles, and freckles

#### P574R

This polymorphism was studied only in a subset of 538 specimens due to the low frequency of the minor allele (0.046), However, the R allele was more frequent among patients with melanomas in the extremities (p = 0.01, p_adjusted _= 0.02; Table 4).

#### R668Q

The tendency to tan or sunburn was associated with the 668RR genotype, which is more frequent among patients who tend to sunburn (p = 0.01, p_adjusted _= 0.02; Table 4).

Significance was not reached when we looked at associations between any of the *MMP-9 *polymorphisms studied and: tumor thickness, tumor infiltrating lymphocytes, number of moles, presence of multiple primary melanoma, lymphovascular invasion, perineural invasion, tumor mitotic index, ulceration, regression, satellites, and distant metastasis.

No associations were found when we performed the Kaplan-Meier analysis for progression, survival and recurrence for all the polymorphisms (data not shown).

#### F571V

We found a previously unreported variation in codon 571 in one sample. The T to G change results in a mutation from F to V (ss49785039). The bulky side-chain of F571 is tightly packed within the hydrophobic core of the MMP-9 hemopexin domain. The substitution of the phenylalanine to valine, a small hydrophobic residue, should not result in steric clashes, destruction of the hydrophobic core or destabilization of beta-strand structure; therefore we evaluate this mutation as a benign change. Conversely, PolyPhen ranked this mutation as "probably damaging".

### Haplotype analysis

The haplotype analysis was performed with the following polymorphisms: promoter (-)1562 C/T, promoter (-)131 (CA)_n_, Q279R and R668Q. The other SNPs were excluded because of their low or null heterozygosity.

The analysis revealed that the most frequent haplotype contained all reference alleles and was (-)1562C-(CA)_< 21_-279Q-668R with a frequency of 55.4%; followed by (-)1562C-(CA)_≥ 21_-279R-668R (18.4%), and (-)1562T- (CA)_≥ 21_-279R-668Q (12.6%). All other haplotypes showed a frequency less than 9%. The Omnibus test showed non-significant differences between clinical stage at diagnosis, Clark level, tumor thickness, tumor site, and phenotypic index, and the MMP-9 haplotypes (data not shown).

## Discussion

There are several reports showing different MMP expression patterns in melanoma [6,33-35] but, to the best of our knowledge, this is the first genetic study to examine the role of polymorphisms in *MMP-9 *in melanoma progression and other melanoma risk factors. We hypothesized that the *MMP-9 *polymorphisms might alter the expression and activity of the enzyme, increasing ECM degradation and invasion, leading to melanoma progression.

Gene transcription is the primary point of regulation of MMPs; therefore, sequence changes in the promoter may have important implications for the transcription, and in turn, for the protein levels and cell physiology. This led us to assess previously described functional polymorphisms within the promoter region of *MMP-9*: (-)1562 C > T and (-)131 (CA)_n_.

The polymorphism at position (-)1562 changes the promoter activity of *MMP-9 *because the T allele abolishes a binding site for a transcription repressor [36]. Although the less active C allele was expected to be present in less aggressive lesions, we found the opposite results. A similar association between this SNP and breast cancer was found by Grieu *et al*, where the T allele correlated with non-ductal histology, positive estrogen receptor and absence of TP53 mutations in breast cancer [37]. However, Matsumura *et al *found significant associations between this allele and the invasive phenotype of gastric cancer [38]. These divergent results indicate that there might be other factors that play a role in the regulation of the *MMP-9 *transcription and/or activity, such as other regulatory elements, promoter methylation or even other steps such as secretion and type of cells under study [39,40].

The dinucleotide repeat shows a bimodal distribution (reviewed by Van den Steen *et al *[15]), with the most prevalent allele being (CA)_14 _and a second peak at (CA)_21–23 _in American white, Finnish, Swedish, Belgian, African-American and southern English population. On the other hand, the Japanese population show the highest incidence of (CA)_21 _followed by (CA)_> 21_. Similar to the SNP at position (-)1562, the length of the microsatellite may influence the transcriptional activity of the gene due to its close localization to the transcriptional start site and several transcription factor binding sites and its length-dependent interaction with nuclear proteins [31]. However, data are inconsistent with regard to the relationship between the length of the microsatellite and the promoter activity [30-32]. Our analysis showed the same distribution and that short alleles were present more frequently in patients at higher risk for melanoma.

There are no published data on the effect of the coding polymorphisms in the MMP-9 activity therefore; it is not possible to hypothesize which variants could be associated with advanced stages of the disease and/or shorter progression-free survival. In this analysis, we found that the reference alleles were more frequent among patients with higher risk for melanoma development (tendency to sunburn, family history of melanoma), more advanced disease (higher melanoma stage, presence of intransit metastasis), and desmoplastic melanoma. However, after adjustment for age, sex, phenotypic index, moles, and freckles only four of these associations remained significant. The 279QQ genotype was associated with the presence of intransit metastasis (p_adjusted _= 0.02) and tendency to sunburn (p_adjusted_< 0.01); the 574R allele was more common among patients with melanomas in the extremities (p_adjusted _= 0.02); and 668RR was associated with the tendency to sunburn (p_adjusted _= 0.02). Our genotyping results suggest that the "reference" MMP-9 enzyme is more active than the "variant" product. These observations agree with the results of a very recent case-control study conducted in Japanese that suggests a link between the R279Q polymorphism and malignant potential of renal cell carcinoma [41].

Our study subjects included ten patients who were younger than 18 years old at diagnosis. In this particular group, the etiology of the disease may differ from the etiology of melanoma in adults. However, other than the presence of spitzoid melanomas in 4 of 10 of these patients, we did not find any additional withstanding clinicopathological or genotypic characteristic and therefore, they were not excluded from the analysis.

Interestingly, variant alleles (-)1562T and 668Q were more frequent among patients with primary melanomas localized to non-cutaneous sites, mainly mucosal melanomas (90%). Whether there is a significant association between these SNPs and mucosal melanoma remains to be determined in a larger group of patients with this type of the disease.

## Conclusion

We examined 437 correlations between clinicopathological variables and 5 *MMP-9 *polymorphisms. Although we found 39 statistically significant associations, after adjustment, only 4 of these associations remained significant and displayed no clear pattern, consequently the correlations may simply be due to chance. Thus, we conclude that this study does not provide strong evidence for further investigation into the role of the *MMP-9 *variants in melanoma progression.

## Abbreviations

CMM, cutaneous malignant melanoma; dbSNP, SNP database from the NCBI; ECM, extracellular matrix; MMP, matrix metalloproteinase; SNP, single nucleotide polymorphism; TIMP, tissue inhibitor of metalloproteinases

## Competing interests

The author(s) declare that they have no competing interests.

## Authors' contributions

JC carried out the genotyping, participated in the selection of SNPs, analysis, and prepared the manuscript; BR carried out the selection of SNPs by *in-silico *methods, contributed to the methods and results section and participated in discussions; NM, NI and SC performed the statistical analysis and contributed to the materials and methods section; AP coordinated the patients' accrual and updated the clinicopathological and epidemiological database; SS and GV participated in the genotyping; DC, KB, AH and AH contributed with subject accrual, pathology review, discussions, and manuscript review; CS participated in the *in-silico *analysis, study design, discussions, and manuscript review; MB conceived and participated in the design of the study, discussions, and review of the manuscript; IO conceived and coordinated the study, participated in its design, analysis, discussion of results, and in the preparation of the manuscript. All authors read and approved the final manuscript.

## Pre-publication history

The pre-publication history for this paper can be accessed here:



## Supplementary Material

Additional File 1 Oligonucleotides and PCR conditions. This table shows PCR primers and PCR conditions for the amplification of the target MMP-9 fragments Click here for file

Additional file 2 Genotyping and statistical analysis for SNPs – individual genotypes. The data shows the statistical analysis of the genotype frequencies for all SNPs and all variables studied Click here for file

Additional file 3 Genotyping and statistical analysis for SNPs – grouped genotypes. The file contains the statistical analysis of the genotype frequencies for all SNPs and all variables studied when grouping genotypes having at least one variant allele or genotypes having at least one reference allele. Click here for file

Additional file 4 Genotyping and statistical analysis for microsatellite. The table shows the statistical analysis of the genotype frequencies for the microsatellite and all variables studied Click here for file
