# Supplementary material for: Matrix Metalloproteinase-9 (MMP-9) polymorphisms in patients with cutaneous malignant melanoma
Source: BMC Med Genet. 2007 Mar 8;8:10. doi: 10.1186/1471-2350-8-10 (PMC1831467; doi:10.1186/1471-2350-8-10)
Supplement: Additional File 1 — Oligonucleotides and PCR conditions. This table shows PCR primers and PCR conditions for the amplification of the target MMP-9 fragments [file 1471-2350-8-10-S1.pdf]

Supplementary Table 1: Oligonucleotides and PCR conditions

Method	Marker	PCR primers		LT probes /PSQ primers (5' à 3')	Ta (ºC)§	PCR additives
		Forward (5' → 3')	Reverse (5' → 3')			
PSQ	(-)1562 (400bp)	GCACATAGTAGGCCCTTTA	TCTATATTCACCTTCTTCAAAGC		56	
	(-)1562 (151bp) £	*Biot-* GGCAGATCACTTGAGTC	GGTTCAAGCAATTCTCCT	CCGAGTAGCTGGTATTATAG	59-0.2 / 57	
	Exon 6 c279	CTTCTCCCCCTTTCCCACATC	*Biot-* TGGCAGGGTTTCCCATCAG	CCCCAGGACTCTACAC	64	
	Exon 12 c668	*Biot-* TGGACACGCACGACGTCTT	GCTTTTTCTTCCTCGCTCAGAAT	CCTCAGCCCTCACCT	58	
LT	Exon 3 c127	GCTCCAGCCTTTCACTTCT	CTCCTCACGTTCTCACCC	AGGATCCAAAACTACTCGGAA-*SPC-Flo*	54	Betaine 1M
	Exon 3 c165	GCTCCAGCCTTTCACTTCT	CTCCTCACGTTCTCACCC	GGACGCAAACATCGTCATCCAGT-*SPC-Flo*	54	Betaine 1M
FA	(-)131 (CA)n	*Flo-* ACTTGGCAGTGGAGACTGC	TGTTGTGGGGGCTTTAAGGAG		58-0.5 / 55	
HET	Exon 10 c574	GTTCAAGGATGGGTGAGGAG	CAGGGGGCGTATTTTTCTAA		55	

Abbreviations: SEQ, sequencing; PSQ, pyrosequencing; LT, LightTyper; FA, fragment size analysis; HET, heteroduplex analysis by DHPLC; Biot, biotin; Flo, fluorescein; SPC, Simple Probe Chemistry; Ta, annealing temperature

£The 151bp fragment is product of a nested PCR (see methods)

§touch-down PCRs are expressed as: initial Ta - ºC per cycle / final Ta
