# Supplementary material for: Matrix Metalloproteinase-9 (MMP-9) polymorphisms in patients with cutaneous malignant melanoma
Source: BMC Med Genet. 2007 Mar 8;8:10. doi: 10.1186/1471-2350-8-10 (PMC1831467; doi:10.1186/1471-2350-8-10)
Supplement: Additional file 2 — Genotyping and statistical analysis for SNPs – individual genotypes. The data shows the statistical analysis of the genotype frequencies for all SNPs and all variables studied [file 1471-2350-8-10-S2.pdf]

Supplementary Table 2: Genotyping and statistical results for SNPs - individual genotypes

	Polymorphism	variable	Homozygote Reference	Heterozygous	Homozygoute Variant	p-value	Test
**Stage at Diagnosis**	MMP9_1562	0	38 (66%)	17 (29%)	3 (5%)		
	MMP9_1562	I	352 (71%)	138 (28%)	9 (2%)		
	MMP9_1562	II	170 (73%)	62 (27%)	1 (0%)		
	MMP9_1562	III	128 (77%)	36 (22%)	3 (2%)		
	MMP9_1562	IV	8 (89%)	1 (11%)	0 (0%)	0.2	Trend
**Current Stage**	MMP9_1562	0	36 (64%)	17 (30%)	3 (5%)		
	MMP9_1562	I	300 (70%)	123 (29%)	8 (2%)		
	MMP9_1562	II	118 (75%)	40 (25%)	0 (0%)		
	MMP9_1562	III	159 (74%)	51 (24%)	4 (2%)		
	MMP9_1562	IV	100 (78%)	26 (20%)	2 (2%)	0.12	Trend
**Thickness**	MMP9_1562	in situ	38 (66%)	17 (29%)	3 (5%)		
	MMP9_1562	<1.01	218 (69%)	93 (29%)	6 (2%)		
	MMP9_1562	1.01 - 2.00	203 (75%)	65 (24%)	3 (1%)		
	MMP9_1562	2.01 - 4.00	118 (74%)	38 (24%)	3 (2%)		
	MMP9_1562	>4.00	85 (71%)	33 (28%)	1 (1%)	0.36	Trend
**Clark Level**	MMP9_1562	I = in situ	38 (66%)	17 (29%)	3 (5%)		
	MMP9_1562	II	74 (69%)	31 (29%)	2 (2%)		
	MMP9_1562	III	99 (67%)	45 (30%)	4 (3%)		
	MMP9_1562	IV	355 (74%)	123 (26%)	5 (1%)		
	MMP9_1562	V	46 (67%)	22 (32%)	1 (1%)	0.32	Trend
**Tumor Infiltrating Lymphocytes**	MMP9_1562	absent	148 (73%)	52 (26%)	4 (2%)		
	MMP9_1562	non-brisk	281 (72%)	107 (27%)	3 (1%)		
	MMP9_1562	brisk	23 (74%)	8 (26%)	0 (0%)	0.68	Trend
**Number of Moles**	MMP9_1562	none	187 (72%)	66 (26%)	6 (2%)		
	MMP9_1562	few	358 (73%)	127 (26%)	8 (2%)		
	MMP9_1562	moderate	116 (70%)	48 (29%)	2 (1%)		
	MMP9_1562	many	29 (73%)	10 (25%)	1 (3%)	0.96	Trend
**Number of Freckles**	MMP9_1562	1 = none	340 (71%)	130 (27%)	6 (1%)		
	MMP9_1562	2	183 (77%)	54 (23%)	2 (1%)		
	MMP9_1562	3	121 (71%)	44 (26%)	5 (3%)		
	MMP9_1562	4	43 (66%)	19 (29%)	3 (5%)		
	MMP9_1562	5	12 (71%)	5 (29%)	0 (0%)		
	MMP9_1562	6 = many	4 (67%)	2 (33%)	0 (0%)	0.44	Trend
**Phenotypic Index**	MMP9_1562	1 = low risk	24 (63%)	14 (37%)	0 (0%)		
	MMP9_1562	2	159 (76%)	48 (23%)	1 (1%)		
	MMP9_1562	3	220 (70%)	85 (27%)	8 (3%)		
	MMP9_1562	4	241 (74%)	80 (25%)	5 (2%)		
	MMP9_1562	5 = high risk	71 (68%)	30 (29%)	3 (3%)	0.32	Trend
**Sex**	MMP9_1562	F	310 (73%)	107 (25%)	8 (2%)		
	MMP9_1562	M	406 (72%)	153 (27%)	9 (2%)	0.72	Chi-Square
**Family History**	MMP9_1562	N	599 (73%)	207 (25%)	13 (2%)		
	MMP9_1562	Y	112 (68%)	49 (30%)	4 (2%)	0.16	Chi-Square
**Multiple Primary**	MMP9_1562	N	614 (73%)	214 (25%)	15 (2%)		
	MMP9_1562	Y	102 (69%)	45 (30%)	2 (1%)	0.36	Chi-Square
**Dysplastic Nevus**	MMP9_1562	N	396 (73%)	136 (25%)	9 (2%)		
	MMP9_1562	Y	143 (66%)	69 (32%)	4 (2%)	0.08	Chi-Square
**Ulceration**	MMP9_1562	absent	379 (71%)	145 (27%)	9 (2%)		
	MMP9_1562	present	138 (74%)	47 (25%)	2 (1%)	0.44	Chi-Square
**Regression**	MMP9_1562	absent	270 (72%)	100 (27%)	3 (1%)		
	MMP9_1562	present	184 (71%)	71 (27%)	4 (2%)	0.6	Chi-Square
**Lymphovascular Invasion**	MMP9_1562	absent	404 (72%)	152 (27%)	7 (1%)		
	MMP9_1562	present	50 (74%)	17 (25%)	1 (2%)	0.8	Chi-Square
**Perineural Invasion**	MMP9_1562	absent	306 (72%)	112 (27%)	5 (1%)		
	MMP9_1562	present	42 (68%)	20 (32%)	0 (0%)	0.6	Chi-Square
**Mitotic Index**	MMP9_1562	absent	73 (72%)	27 (27%)	2 (2%)		
	MMP9_1562	present	327 (72%)	125 (27%)	4 (1%)	0.8	Chi-Square
**Satellites**	MMP9_1562	absent	191 (76%)	59 (24%)	1 (0%)		
	MMP9_1562	present	25 (83%)	5 (17%)	0 (0%)	0.36	Chi-Square
**Solar Elastosis**	MMP9_1562	absent	18 (82%)	4 (18%)	0 (0%)		
	MMP9_1562	present	31 (78%)	9 (23%)	0 (0%)	0.68	Chi-Square
**Distant Metastasis**	MMP9_1562	N	615 (71%)	232 (27%)	15 (2%)		

	Polymorphism	variable	Homozygote Reference	Heterozygous	Homozygoute Variant	p-value	Test
	MMP9_1562	Y	101 (78%)	26 (20%)	2 (2%)	0.12	Chi-Square
**Intransit Metastasis**	MMP9_1562	N	668 (72%)	249 (27%)	16 (2%)		
	MMP9_1562	Y	21 (78%)	5 (19%)	1 (4%)	0.68	Chi-Square
**Tan/Burn Index**	MMP9_1562	tend to tan	59 (65%)	30 (33%)	2 (2%)		
	MMP9_1562	tend to sunburn	657 (73%)	230 (25%)	15 (2%)	0.12	Chi-Square
**Race**	MMP9_1562	White non-Hispanic	682 (72%)	254 (27%)	16 (2%)		
	MMP9_1562	Hispanic	9 (82%)	2 (18%)	0 (0%)		
	MMP9_1562	Black non-Hispanic	10 (91%)	0 (0%)	1 (9%)		
	MMP9_1562	Asian/Indian	2 (67%)	1 (33%)	0 (0%)	0.28	Chi-Square
**Site**	MMP9_1562	extremities	378 (71%)	145 (27%)	10 (2%)		
	MMP9_1562	head & neck	49 (69%)	21 (30%)	1 (1%)		
	MMP9_1562	non-cutaneous	6 (55%)	3 (27%)	2 (18%)		
	MMP9_1562	trunk	249 (74%)	83 (25%)	4 (1%)	<0.01	Chi-Square
**Histology**	MMP9_1562	desmoplastic	17 (74%)	6 (26%)	0 (0%)		
	MMP9_1562	other	171 (71%)	65 (27%)	4 (2%)		
	MMP9_1562	spitzoid	8 (80%)	2 (20%)	0 (0%)		
	MMP9_1562	unknown	520 (72%)	187 (26%)	13 (2%)	1	Chi-Square
**Stage at Diagnosis**	MMP9_279	0	27 (47%)	22 (38%)	9 (16%)		
	MMP9_279	I	200 (40%)	232 (46%)	72 (14%)		
	MMP9_279	II	91 (39%)	117 (50%)	26 (11%)		
	MMP9_279	III	82 (49%)	67 (40%)	20 (12%)		
	MMP9_279	IV	4 (44%)	5 (56%)	0 (0%)	0.32	Trend
**Current Stage**	MMP9_279	0	25 (45%)	22 (39%)	9 (16%)		
	MMP9_279	I	174 (40%)	199 (46%)	62 (14%)		
	MMP9_279	II	60 (38%)	84 (53%)	15 (9%)		
	MMP9_279	III	99 (46%)	92 (43%)	25 (12%)		
	MMP9_279	IV	59 (46%)	52 (40%)	18 (14%)	0.36	Trend
**Thickness**	MMP9_279	in situ	27 (47%)	22 (38%)	9 (16%)		
	MMP9_279	<1.01	127 (40%)	149 (46%)	45 (14%)		
	MMP9_279	1.01 - 2.00	117 (43%)	117 (43%)	38 (14%)		
	MMP9_279	2.01 - 4.00	65 (41%)	74 (46%)	21 (13%)		
	MMP9_279	>4.00	43 (36%)	67 (55%)	11 (9%)	0.48	Trend
**Clark Level**	MMP9_279	I = in situ	27 (47%)	22 (38%)	9 (16%)		
	MMP9_279	II	38 (35%)	57 (53%)	13 (12%)		
	MMP9_279	III	64 (43%)	65 (43%)	21 (14%)		
	MMP9_279	IV	194 (40%)	227 (47%)	67 (14%)		
	MMP9_279	V	26 (38%)	36 (52%)	7 (10%)	0.72	Trend
**Tumor Infiltrating Lymphocytes**	MMP9_279	absent	89 (43%)	98 (48%)	19 (9%)		
	MMP9_279	non-brisk	155 (39%)	195 (49%)	46 (12%)		
	MMP9_279	brisk	14 (45%)	13 (42%)	4 (13%)	0.76	Trend
**Number of Moles**	MMP9_279	none	117 (45%)	107 (41%)	36 (14%)		
	MMP9_279	few	201 (40%)	232 (47%)	64 (13%)		
	MMP9_279	moderate	69 (41%)	78 (46%)	22 (13%)		
	MMP9_279	many	17 (43%)	18 (45%)	5 (13%)	0.88	Trend
**Number of Freckles**	MMP9_279	1 = none	198 (41%)	222 (46%)	61 (13%)		
	MMP9_279	2	114 (48%)	100 (42%)	26 (11%)		
	MMP9_279	3	70 (41%)	77 (45%)	25 (15%)		
	MMP9_279	4	23 (35%)	29 (45%)	13 (20%)		
	MMP9_279	5	6 (35%)	10 (59%)	1 (6%)		
	MMP9_279	6 = many	3 (50%)	3 (50%)	0 (0%)	0.48	Trend
**Phenotypic Index**	MMP9_279	1 = low risk	8 (21%)	23 (61%)	7 (18%)		
	MMP9_279	2	99 (47%)	84 (40%)	27 (13%)		
	MMP9_279	3	129 (41%)	143 (45%)	44 (14%)		
	MMP9_279	4	145 (44%)	149 (45%)	34 (10%)		
	MMP9_279	5 = high risk	39 (37%)	49 (47%)	17 (16%)	0.12	Trend
**Sex**	MMP9_279	F	185 (43%)	192 (45%)	52 (12%)		
	MMP9_279	M	235 (41%)	259 (45%)	78 (14%)	0.4	Chi-Square
**Family History**	MMP9_279	N	352 (43%)	368 (45%)	105 (13%)		
	MMP9_279	Y	65 (39%)	78 (47%)	24 (14%)	0.36	Chi-Square
**Multiple Primary**	MMP9_279	N	361 (43%)	379 (45%)	109 (13%)		
	MMP9_279	Y	59 (39%)	71 (47%)	21 (14%)	0.44	Chi-Square
**Dysplastic Nevus**	MMP9_279	N	239 (44%)	235 (43%)	69 (13%)		
	MMP9_279	Y	80 (37%)	103 (47%)	35 (16%)	0.04	Chi-Square
**Ulceration**	MMP9_279	absent	213 (40%)	254 (47%)	70 (13%)		

	Polymorphism	variable	Homozygote Reference	Heterozygous	Homozygoute Variant	p-value	Test
	MMP9_279	present	77 (41%)	89 (47%)	24 (13%)	0.84	Chi-Square
**Regression**	MMP9_279	absent	165 (44%)	174 (46%)	38 (10%)		
	MMP9_279	present	100 (38%)	130 (50%)	32 (12%)	0.16	Chi-Square
**Lymphovascular Invasion**	MMP9_279	absent	230 (40%)	271 (48%)	68 (12%)		
	MMP9_279	present	28 (41%)	33 (49%)	7 (10%)	0.76	Chi-Square
**Perineural Invasion**	MMP9_279	absent	174 (41%)	202 (47%)	52 (12%)		
	MMP9_279	present	25 (40%)	32 (52%)	5 (8%)	0.68	Chi-Square
**Mitotic Index**	MMP9_279	absent	42 (41%)	48 (47%)	13 (13%)		
	MMP9_279	present	188 (41%)	220 (48%)	53 (12%)	0.88	Chi-Square
**Satellites**	MMP9_279	absent	110 (43%)	126 (50%)	18 (7%)		
	MMP9_279	present	13 (43%)	13 (43%)	4 (13%)	0.6	Chi-Square
**Solar Elastosis**	MMP9_279	absent	15 (68%)	7 (32%)	0 (0%)		
	MMP9_279	present	15 (38%)	21 (53%)	4 (10%)	<0.01	Chi-Square
**Distant Metastasis**	MMP9_279	N	360 (41%)	397 (46%)	112 (13%)		
	MMP9_279	Y	60 (46%)	52 (40%)	18 (14%)	0.56	Chi-Square
**Intransit Metastasis**	MMP9_279	N	384 (41%)	435 (46%)	122 (13%)		
	MMP9_279	Y	17 (63%)	7 (26%)	3 (11%)	0.08	Chi-Square
**Tan/Burn Index**	MMP9_279	tend to tan	26 (28%)	48 (53%)	17 (19%)		
	MMP9_279	tend to sunburn	394 (43%)	403 (44%)	113 (13%)	<0.01	Chi-Square
**Race**	MMP9_279	White non-Hispanic	401 (42%)	432 (45%)	127 (13%)		
	MMP9_279	Hispanic	6 (55%)	5 (46%)	0 (0%)		
	MMP9_279	Black non-Hispanic	5 (46%)	5 (46%)	1 (9%)		
	MMP9_279	Asian/Indian	1 (33%)	2 (67%)	0 (0%)	0.84	Chi-Square
**Site**	MMP9_279	extremities	212 (40%)	248 (46%)	75 (14%)		
	MMP9_279	head & neck	25 (35%)	30 (42%)	16 (23%)		
	MMP9_279	non-cutaneous	6 (50%)	4 (33%)	2 (17%)		
	MMP9_279	trunk	152 (45%)	155 (46%)	34 (10%)	0.12	Chi-Square
**Histology**	MMP9_279	desmoplastic	7 (30%)	12 (52%)	4 (17%)		
	MMP9_279	other	91 (37%)	122 (50%)	31 (13%)		
	MMP9_279	spitzoid	3 (30%)	6 (60%)	1 (10%)		
	MMP9_279	unknown	319 (44%)	311 (43%)	94 (13%)	0.4	Chi-Square
**Stage at Diagnosis**	MMP9_574	0	28 (90%)	2 (7%)	1 (3%)		
	MMP9_574	I	257 (92%)	21 (8%)	0 (0%)		
	MMP9_574	II	100 (89%)	13 (12%)	0 (0%)		
	MMP9_574	III	84 (89%)	10 (11%)	0 (0%)		
	MMP9_574	IV	3 (75%)	1 (25%)	0 (0%)	<0.01	Trend
**Current Stage**	MMP9_574	0	27 (90%)	2 (7%)	1 (3%)		
	MMP9_574	I	221 (92%)	19 (8%)	0 (0%)		
	MMP9_574	II	68 (88%)	9 (12%)	0 (0%)		
	MMP9_574	III	87 (90%)	10 (10%)	0 (0%)		
	MMP9_574	IV	79 (92%)	7 (8%)	0 (0%)	0.04	Trend
**Thickness**	MMP9_574	in situ	28 (90%)	2 (7%)	1 (3%)		
	MMP9_574	<1.01	172 (93%)	14 (8%)	0 (0%)		
	MMP9_574	1.01 - 2.00	126 (93%)	10 (7%)	0 (0%)		
	MMP9_574	2.01 - 4.00	68 (85%)	12 (15%)	0 (0%)		
	MMP9_574	>4.00	59 (92%)	5 (8%)	0 (0%)	<0.01	Trend
**Clark Level**	MMP9_574	I = in situ	28 (90%)	2 (7%)	1 (3%)		
	MMP9_574	II	62 (93%)	5 (8%)	0 (0%)		
	MMP9_574	III	79 (93%)	6 (7%)	0 (0%)		
	MMP9_574	IV	217 (90%)	24 (10%)	0 (0%)		
	MMP9_574	V	34 (87%)	5 (13%)	0 (0%)	0.04	Trend
**Tumor Infiltrating Lymphocytes**	MMP9_574	absent	106 (89%)	13 (11%)	0 (0%)		
	MMP9_574	non-brisk	162 (90%)	18 (10%)	0 (0%)		
	MMP9_574	brisk	15 (94%)	1 (6%)	0 (0%)	0.84	Trend
**Number of Moles**	MMP9_574	none	153 (92%)	13 (8%)	1 (1%)		
	MMP9_574	few	211 (91%)	22 (9%)	0 (0%)		
	MMP9_574	moderate	72 (95%)	4 (5%)	0 (0%)		
	MMP9_574	many	24 (86%)	4 (14%)	0 (0%)	0.6	Trend
**Number of Freckles**	MMP9_574	1 = none	227 (90%)	24 (10%)	1 (0%)		
	MMP9_574	2	124 (89%)	15 (11%)	0 (0%)		
	MMP9_574	3	81 (93%)	6 (7%)	0 (0%)		
	MMP9_574	4	32 (100%)	0 (0%)	0 (0%)		
	MMP9_574	5	6 (100%)	0 (0%)	0 (0%)		
	MMP9_574	6 = many	1 (50%)	1 (50%)	0 (0%)	0.44	Trend

	Polymorphism	variable	Homozygote Reference	Heterozygous	Homozygoute Variant	p-value	Test
**Phenotypic Index**	MMP9_574	1 = low risk	15 (75%)	4 (20%)	1 (5%)		
	MMP9_574	2	87 (88%)	12 (12%)	0 (0%)		
	MMP9_574	3	165 (95%)	8 (5%)	0 (0%)		
	MMP9_574	4	161 (90%)	17 (10%)	0 (0%)		
	MMP9_574	5 = high risk	56 (90%)	6 (10%)	0 (0%)	<0.01	Chi-Square
**Sex**	MMP9_574	F	220 (91%)	21 (9%)	1 (0%)		
	MMP9_574	M	265 (91%)	26 (9%)	0 (0%)	0.84	Chi-Square
**Family History**	MMP9_574	N	395 (90%)	45 (10%)	0 (0%)		
	MMP9_574	Y	88 (97%)	2 (2%)	1 (1%)	0.08	Chi-Square
**Multiple Primary**	MMP9_574	N	410 (90%)	44 (10%)	1 (0%)		
	MMP9_574	Y	75 (96%)	3 (4%)	0 (0%)	0.08	Chi-Square
**Dysplastic Nevus**	MMP9_574	N	307 (90%)	32 (9%)	1 (0%)		
	MMP9_574	Y	117 (93%)	9 (7%)	0 (0%)	0.36	Chi-Square
**Ulceration**	MMP9_574	absent	254 (91%)	25 (9%)	0 (0%)		
	MMP9_574	present	85 (89%)	11 (12%)	0 (0%)	0.48	Chi-Square
**Regression**	MMP9_574	absent	181 (90%)	20 (10%)	0 (0%)		
	MMP9_574	present	104 (90%)	12 (10%)	0 (0%)	0.92	Chi-Square
**Lymphovascular Invasion**	MMP9_574	absent	267 (90%)	30 (10%)	0 (0%)		
	MMP9_574	present	26 (93%)	2 (7%)	0 (0%)	0.6	Chi-Square
**Perineural Invasion**	MMP9_574	absent	176 (90%)	20 (10%)	0 (0%)		
	MMP9_574	present	26 (90%)	3 (10%)	0 (0%)	1	Chi-Square
**Mitotic Index**	MMP9_574	absent	58 (92%)	5 (8%)	0 (0%)		
	MMP9_574	present	184 (89%)	23 (11%)	0 (0%)	0.48	Chi-Square
**Satellites**	MMP9_574	absent	116 (89%)	14 (11%)	0 (0%)		
	MMP9_574	present	14 (93%)	1 (7%)	0 (0%)	0.64	Chi-Square
**Solar Elastosis**	MMP9_574	absent	11 (92%)	1 (8%)	0 (0%)		
	MMP9_574	present	22 (85%)	4 (15%)	0 (0%)	0.56	Chi-Square
**Distant Metastasis**	MMP9_574	N	405 (91%)	40 (9%)	1 (0%)		
	MMP9_574	Y	79 (92%)	7 (8%)	0 (0%)	0.72	Chi-Square
**Intransit Metastasis**	MMP9_574	N	449 (92%)	39 (8%)	1 (0%)		
	MMP9_574	Y	19 (91%)	2 (10%)	0 (0%)	0.84	Chi-Square
**Tan/Burn Index**	MMP9_574	tend to tan	45 (90%)	4 (8%)	1 (2%)		
	MMP9_574	tend to sunburn	440 (91%)	43 (9%)	0 (0%)	0.48	Chi-Square
**Race**	MMP9_574	White non-Hispanic	465 (92%)	42 (8%)	1 (0%)		
	MMP9_574	Hispanic	5 (63%)	3 (38%)	0 (0%)		
	MMP9_574	Black non-Hispanic	4 (67%)	2 (33%)	0 (0%)		
	MMP9_574	Asian/Indian	2 (100%)	0 (0%)	0 (0%)	0.04	Chi-Square
**Site**	MMP9_574	extremities	263 (88%)	36 (12%)	0 (0%)		
	MMP9_574	head & neck	36 (97%)	1 (3%)	0 (0%)		
	MMP9_574	non-cutaneous	6 (100%)	0 (0%)	0 (0%)		
	MMP9_574	trunk	164 (96%)	6 (4%)	1 (1%)	0.04	Chi-Square
**Histology**	MMP9_574	desmoplastic	7 (88%)	1 (13%)	0 (0%)		
	MMP9_574	other	117 (91%)	12 (9%)	0 (0%)		
	MMP9_574	spitzoid	2 (50%)	2 (50%)	0 (0%)		
	MMP9_574	unknown	359 (92%)	32 (8%)	1 (0%)	0.16	Chi-Square
**Stage at Diagnosis**	MMP9_668	0	38 (66%)	18 (31%)	2 (3%)		
	MMP9_668	I	355 (71%)	135 (27%)	12 (2%)		
	MMP9_668	II	164 (70%)	68 (29%)	1 (0%)		
	MMP9_668	III	130 (77%)	35 (21%)	3 (2%)		
	MMP9_668	IV	8 (89%)	1 (11%)	0 (0%)	0.28	Trend
**Current Stage**	MMP9_668	0	36 (64%)	18 (32%)	2 (4%)		
	MMP9_668	I	302 (70%)	120 (28%)	11 (3%)		
	MMP9_668	II	114 (72%)	44 (28%)	0 (0%)		
	MMP9_668	III	163 (76%)	49 (23%)	4 (2%)		
	MMP9_668	IV	98 (77%)	28 (22%)	2 (2%)	0.28	Trend
**Thickness**	MMP9_668	in situ	38 (66%)	18 (31%)	2 (3%)		
	MMP9_668	<1.01	219 (69%)	91 (29%)	9 (3%)		
	MMP9_668	1.01 - 2.00	205 (75%)	64 (24%)	3 (1%)		
	MMP9_668	2.01 - 4.00	115 (72%)	41 (26%)	3 (2%)		
	MMP9_668	>4.00	84 (70%)	35 (29%)	1 (1%)	0.52	Trend
**Clark Level**	MMP9_668	I = in situ	38 (66%)	18 (31%)	2 (3%)		
	MMP9_668	II	73 (68%)	32 (30%)	2 (2%)		
	MMP9_668	III	101 (67%)	42 (28%)	7 (5%)		
	MMP9_668	IV	355 (73%)	125 (26%)	5 (1%)		

	Polymorphism	variable	Homozygote Reference	Heterozygous	Homozygoute Variant	p-value	Test
	MMP9_668	V	45 (65%)	23 (33%)	1 (1%)	0.16	Trend
**Tumor Infiltrating Lymphocytes**	MMP9_668	absent	146 (71%)	55 (27%)	4 (2%)		
	MMP9_668	non-brisk	279 (71%)	109 (28%)	5 (1%)		
	MMP9_668	brisk	24 (77%)	7 (23%)	0 (0%)	0.84	Trend
**Number of Moles**	MMP9_668	none	183 (71%)	68 (26%)	8 (3%)		
	MMP9_668	few	359 (72%)	128 (26%)	9 (2%)		
	MMP9_668	moderate	119 (71%)	47 (28%)	1 (1%)		
	MMP9_668	many	30 (75%)	9 (23%)	1 (3%)	0.68	Trend
**Number of Freckles**	MMP9_668	1 = none	338 (71%)	134 (28%)	6 (1%)		
	MMP9_668	2	183 (76%)	54 (23%)	3 (1%)		
	MMP9_668	3	122 (71%)	44 (26%)	5 (3%)		
	MMP9_668	4	44 (68%)	17 (26%)	4 (6%)		
	MMP9_668	5	12 (71%)	5 (29%)	0 (0%)		
	MMP9_668	6 = many	4 (67%)	2 (33%)	0 (0%)	0.24	Trend
**Phenotypic Index**	MMP9_668	1 = low risk	20 (53%)	18 (47%)	0 (0%)		
	MMP9_668	2	160 (77%)	46 (22%)	2 (1%)		
	MMP9_668	3	222 (71%)	83 (26%)	10 (3%)		
	MMP9_668	4	240 (73%)	82 (25%)	5 (2%)		
	MMP9_668	5 = high risk	73 (70%)	30 (29%)	2 (2%)	0.04	Trend
**Sex**	MMP9_668	F	311 (73%)	109 (26%)	7 (2%)		
	MMP9_668	M	405 (71%)	153 (27%)	12 (2%)	0.48	Chi-Square
**Family History**	MMP9_668	N	598 (73%)	211 (26%)	14 (2%)		
	MMP9_668	Y	113 (69%)	47 (29%)	5 (3%)	0.2	Chi-Square
**Multiple Primary**	MMP9_668	N	612 (72%)	218 (26%)	17 (2%)		
	MMP9_668	Y	104 (70%)	43 (29%)	2 (1%)	0.68	Chi-Square
**Dysplastic Nevus**	MMP9_668	N	395 (73%)	137 (25%)	11 (2%)		
	MMP9_668	Y	145 (67%)	68 (31%)	4 (2%)	0.16	Chi-Square
**Ulceration**	MMP9_668	absent	379 (71%)	144 (27%)	12 (2%)		
	MMP9_668	present	135 (72%)	51 (27%)	2 (1%)	0.6	Chi-Square
**Regression**	MMP9_668	absent	271 (72%)	101 (27%)	4 (1%)		
	MMP9_668	present	180 (70%)	74 (29%)	5 (2%)	0.4	Chi-Square
**Lymphovascular Invasion**	MMP9_668	absent	400 (71%)	156 (28%)	9 (2%)		
	MMP9_668	present	50 (74%)	16 (24%)	2 (3%)	0.84	Chi-Square
**Perineural Invasion**	MMP9_668	absent	303 (72%)	114 (27%)	7 (2%)		
	MMP9_668	present	42 (68%)	20 (32%)	0 (0%)	0.76	Chi-Square
**Mitotic Index**	MMP9_668	absent	73 (72%)	27 (27%)	2 (2%)		
	MMP9_668	present	324 (71%)	128 (28%)	6 (1%)	0.96	Chi-Square
**Satellites**	MMP9_668	absent	189 (75%)	62 (25%)	1 (0%)		
	MMP9_668	present	25 (83%)	5 (17%)	0 (0%)	0.32	Chi-Square
**Solar Elastosis**	MMP9_668	absent	18 (82%)	4 (18%)	0 (0%)		
	MMP9_668	present	30 (75%)	10 (25%)	0 (0%)	0.52	Chi-Square
**Distant Metastasis**	MMP9_668	N	617 (71%)	232 (27%)	17 (2%)		
	MMP9_668	Y	99 (77%)	28 (22%)	2 (2%)	0.2	Chi-Square
**Intransit Metastasis**	MMP9_668	N	667 (71%)	252 (27%)	18 (2%)		
	MMP9_668	Y	22 (82%)	4 (15%)	1 (4%)	0.4	Chi-Square
**Tan/Burn Index**	MMP9_668	tend to tan	55 (60%)	34 (37%)	2 (3%)		
	MMP9_668	tend to sunburn	661 (73%)	228 (25%)	17 (2%)	0.02	Chi-Square
**Race**	MMP9_668	White non-Hispanic	687 (72%)	251 (26%)	18 (2%)		
	MMP9_668	Hispanic	7 (64%)	4 (36%)	0 (0%)		
	MMP9_668	Black non-Hispanic	7 (64%)	3 (27%)	1 (9%)		
	MMP9_668	Asian/Indian	2 (67%)	1 (33%)	0 (0%)	0.68	Chi-Square
**Site**	MMP9_668	extremities	375 (70%)	146 (27%)	12 (2%)		
	MMP9_668	head & neck	49 (69%)	21 (30%)	1 (1%)		
	MMP9_668	non-cutaneous	6 (55%)	3 (27%)	2 (18%)		
	MMP9_668	trunk	252 (74%)	84 (25%)	4 (1%)	<0.01	Chi-Square
**Histology**	MMP9_668	desmoplastic	16 (70%)	7 (30%)	0 (0%)		
	MMP9_668	other	170 (70%)	68 (28%)	4 (2%)		
	MMP9_668	spitzoid	8 (80%)	2 (20%)	0 (0%)		
	MMP9_668	unknown	522 (72%)	185 (26%)	15 (2%)	0.96	Chi-Square
