# Supplementary material for: Matrix Metalloproteinase-9 (MMP-9) polymorphisms in patients with cutaneous malignant melanoma
Source: BMC Med Genet. 2007 Mar 8;8:10. doi: 10.1186/1471-2350-8-10 (PMC1831467; doi:10.1186/1471-2350-8-10)
Supplement: Additional file 3 — Genotyping and statistical analysis for SNPs – grouped genotypes. The file contains the statistical analysis of the genotype frequencies for all SNPs and all variables studied when grouping genotypes having at least one variant allele or genotypes having at least one reference allele. [file 1471-2350-8-10-S3.pdf]

**Supplementary Table 3**: Genotyping and statistical results for SNPs - grouped genotypes											
	**Polymorphism**	**variable**	**Homozygote Reference**	**Any Variant**	**p-value**	**Test**		**Homozygote Variant**	**Any Reference**	**p-value**	**Test**
**Stage at Diagnosis**	MMP9_1562	0	38 (66%)	20 (35%)				3 (5%)	55 (95%)		
	MMP9_1562	I	352 (71%)	147 (30%)				9 (2%)	490 (98%)		
	MMP9_1562	II	170 (73%)	63 (27%)				1 (0%)	232 (100%)		
	MMP9_1562	III	128 (77%)	39 (23%)				3 (2%)	164 (98%)		
	MMP9_1562	IV	8 (89%)	1 (11%)	0.04	Trend		0 (0%)	9 (100%)	0.16	Trend
**Current Stage**	MMP9_1562	0	36 (64%)	20 (36%)				3 (5%)	53 (95%)		
	MMP9_1562	I	300 (70%)	131 (30%)				8 (2%)	423 (98%)		
	MMP9_1562	II	118 (75%)	40 (25%)				0 (0%)	158 (100%)		
	MMP9_1562	III	159 (74%)	55 (26%)				4 (2%)	210 (98%)		
	MMP9_1562	IV	100 (78%)	28 (22%)	<0.01	Trend		2 (2%)	126 (98%)	0.32	Trend
**Thickness**	MMP9_1562	in situ	38 (66%)	20 (35%)				3 (5%)	55 (95%)		
	MMP9_1562	<1.01	218 (69%)	99 (31%)				6 (2%)	311 (98%)		
	MMP9_1562	1.01 - 2.00	203 (75%)	68 (25%)				3 (1%)	268 (99%)		
	MMP9_1562	2.01 - 4.00	118 (74%)	41 (26%)				3 (2%)	156 (98%)		
	MMP9_1562	>4.00	85 (71%)	34 (29%)	0.2	Trend		1 (1%)	118 (99%)	0.16	Trend
**Clark Level**	MMP9_1562	I = in situ	38 (66%)	20 (35%)				3 (5%)	55 (95%)		
	MMP9_1562	II	74 (69%)	33 (31%)				2 (2%)	105 (98%)		
	MMP9_1562	III	99 (67%)	49 (33%)				4 (3%)	144 (97%)		
	MMP9_1562	IV	355 (74%)	128 (27%)				5 (1%)	478 (99%)		
	MMP9_1562	V	46 (67%)	23 (33%)	0.28	Trend		1 (1%)	68 (99%)	0.04	Trend
**Tumor Infiltrating Lymphocytes**	MMP9_1562	absent	148 (73%)	56 (28%)				4 (2%)	200 (98%)		
	MMP9_1562	non-brisk	281 (72%)	110 (28%)				3 (1%)	388 (99%)		
	MMP9_1562	brisk	23 (74%)	8 (26%)	1	Trend		0 (0%)	31 (100%)	0.16	Trend
**Number of Moles**	MMP9_1562	none	187 (72%)	72 (28%)				6 (2%)	253 (98%)		
	MMP9_1562	few	358 (73%)	135 (27%)				8 (2%)	485 (98%)		
	MMP9_1562	moderate	116 (70%)	50 (30%)				2 (1%)	164 (99%)		
	MMP9_1562	many	29 (73%)	11 (28%)	0.72	Trend		1 (3%)	39 (98%)	0.56	Trend
**Number of Freckles**	MMP9_1562	1 = none	340 (71%)	136 (29%)				6 (1%)	470 (99%)		
	MMP9_1562	2	183 (77%)	56 (23%)				2 (1%)	237 (99%)		
	MMP9_1562	3	121 (71%)	49 (29%)				5 (3%)	165 (97%)		
	MMP9_1562	4	43 (66%)	22 (34%)				3 (5%)	62 (95%)		
	MMP9_1562	5	12 (71%)	5 (29%)				0 (0%)	17 (100%)		
	MMP9_1562	6 = many	4 (67%)	2 (33%)	0.6	Trend		0 (0%)	6 (100%)	0.12	Trend
**Phenotypic Index**	MMP9_1562	1 = low risk	24 (63%)	14 (37%)				0 (0%)	38 (100%)		
	MMP9_1562	2	159 (76%)	49 (24%)				1 (1%)	207 (100%)		
	MMP9_1562	3	220 (70%)	93 (30%)				8 (3%)	305 (97%)		
	MMP9_1562	4	241 (74%)	85 (26%)				5 (2%)	321 (99%)		
	MMP9_1562	5 = high risk	71 (68%)	33 (32%)	0.76	Trend		3 (3%)	101 (97%)	0.16	Trend
**Sex**	MMP9_1562	F	310 (73%)	115 (27%)				8 (2%)	417 (98%)		
	MMP9_1562	M	406 (72%)	162 (29%)	0.6	Fisher		9 (2%)	559 (98%)	0.8	Fisher
**Family History**	MMP9_1562	N	599 (73%)	220 (27%)				13 (2%)	806 (98%)		
	MMP9_1562	Y	112 (68%)	53 (32%)	0.2	Fisher		4 (2%)	161 (98%)	0.52	Fisher
**Multiple Primary**	MMP9_1562	N	614 (73%)	229 (27%)				15 (2%)	828 (98%)		

	Polymorphism	variable	Homozygote Reference	Any Variant	p-value	Test		Homozygote Variant	Any Reference	p-value	Test
	MMP9_1562	Y	102 (69%)	47 (32%)	0.28	Fisher		2 (1%)	147 (99%)	1	Fisher
**Dysplastic Nevus**	MMP9_1562	N	396 (73%)	145 (27%)				9 (2%)	532 (98%)		
	MMP9_1562	Y	143 (66%)	73 (34%)	0.08	Fisher		4 (2%)	212 (98%)	1	Fisher
**Ulceration**	MMP9_1562	absent	379 (71%)	154 (29%)				9 (2%)	524 (98%)		
	MMP9_1562	present	138 (74%)	49 (26%)	0.52	Fisher		2 (1%)	185 (99%)	0.72	Fisher
**Regression**	MMP9_1562	absent	270 (72%)	103 (28%)				3 (1%)	370 (99%)		
	MMP9_1562	present	184 (71%)	75 (29%)	0.72	Fisher		4 (2%)	255 (99%)	0.44	Fisher
**Lymphovascular Invasion**	MMP9_1562	absent	404 (72%)	159 (28%)				7 (1%)	556 (99%)		
	MMP9_1562	present	50 (74%)	18 (27%)	0.88	Fisher		1 (2%)	67 (99%)	0.6	Fisher
**Perineural Invasion**	MMP9_1562	absent	306 (72%)	117 (28%)				5 (1%)	418 (99%)		
	MMP9_1562	present	42 (68%)	20 (32%)	0.44	Fisher		0 (0%)	62 (100%)	1	Fisher
**Mitotic Index**	MMP9_1562	absent	73 (72%)	29 (28%)				2 (2%)	100 (98%)		
	MMP9_1562	present	327 (72%)	129 (28%)	1	Fisher		4 (1%)	452 (99%)	0.32	Fisher
**Satellites**	MMP9_1562	absent	191 (76%)	60 (24%)				1 (0%)	250 (100%)		
	MMP9_1562	present	25 (83%)	5 (17%)	0.48	Fisher		0 (0%)	30 (100%)	1	Fisher
**Solar Elastosis**	MMP9_1562	absent	18 (82%)	4 (18%)				0 (0%)	22 (100%)		
	MMP9_1562	present	31 (78%)	9 (23%)	0.76	Fisher		0 (0%)	40 (100%)	0.76	Fisher
**Distant Metastasis**	MMP9_1562	N	615 (71%)	247 (29%)				15 (2%)	847 (98%)		
	MMP9_1562	Y	101 (78%)	28 (22%)	0.12	Fisher		2 (2%)	127 (98%)	1	Fisher
**Intransit Metastasis**	MMP9_1562	N	668 (72%)	265 (28%)				16 (2%)	917 (98%)		
	MMP9_1562	Y	21 (78%)	6 (22%)	0.68	Fisher		1 (4%)	26 (96%)	0.4	Fisher
**Tan/Burn Index**	MMP9_1562	tend to tan	59 (65%)	32 (35%)				2 (2%)	89 (98%)		
	MMP9_1562	tend to sunburn	657 (73%)	245 (27%)	0.12	Fisher		15 (2%)	887 (98%)	0.68	Fisher
**Race**	MMP9_1562	White non-Hispanic	682 (72%)	270 (28%)				16 (2%)	936 (98%)		
	MMP9_1562	Hispanic	9 (82%)	2 (18%)				0 (0%)	11 (100%)		
	MMP9_1562	Black non-Hispanic	10 (91%)	1 (9%)				1 (9%)	10 (91%)		
	MMP9_1562	Asian/Indian	2 (67%)	1 (33%)	0.48	Chi-Square		0 (0%)	3 (100%)	0.28	Chi-Square
**Site**	MMP9_1562	extremities	378 (71%)	155 (29%)				10 (2%)	523 (98%)		
	MMP9_1562	head & neck	49 (69%)	22 (31%)				1 (1%)	70 (99%)		
	MMP9_1562	non-cutaneous	6 (55%)	5 (46%)				2 (18%)	9 (82%)		
	MMP9_1562	trunk	249 (74%)	87 (26%)	0.4	Chi-Square		4 (1%)	332 (99%)	<0.01	Chi-Square
**Histology**	MMP9_1562	desmoplastic	17 (74%)	6 (26%)				0 (0%)	23 (100%)		
	MMP9_1562	other	171 (71%)	69 (29%)				4 (2%)	236 (98%)		
	MMP9_1562	spitzoid	8 (80%)	2 (20%)				0 (0%)	10 (100%)		
	MMP9_1562	unknown	520 (72%)	200 (28%)	0.92	Chi-Square		13 (2%)	707 (98%)	0.88	Chi-Square
**Stage at Diagnosis**	MMP9_279	0	27 (47%)	31 (53%)				9 (16%)	49 (85%)		
	MMP9_279	I	200 (40%)	304 (60%)				72 (14%)	432 (86%)		
	MMP9_279	II	91 (39%)	143 (61%)				26 (11%)	208 (89%)		
	MMP9_279	III	82 (49%)	87 (52%)				20 (12%)	149 (88%)		
	MMP9_279	IV	4 (44%)	5 (56%)	0.24	Trend		0 (0%)	9 (100%)	0.16	Trend
**Current Stage**	MMP9_279	0	25 (45%)	31 (55%)				9 (16%)	47 (84%)		
	MMP9_279	I	174 (40%)	261 (60%)				62 (14%)	373 (86%)		
	MMP9_279	II	60 (38%)	99 (62%)				15 (9%)	144 (91%)		
	MMP9_279	III	99 (46%)	117 (54%)				25 (12%)	191 (88%)		
	MMP9_279	IV	59 (46%)	70 (54%)	0.2	Trend		18 (14%)	111 (86%)	0.44	Trend
**Thickness**	MMP9_279	in situ	27 (47%)	31 (53%)				9 (16%)	49 (85%)		

	Polymorphism	variable	Homozygote Reference	Any Variant	p-value	Test		Homozygote Variant	Any Reference	p-value	Test
	MMP9_279	<1.01	127 (40%)	194 (60%)				45 (14%)	276 (86%)		
	MMP9_279	1.01 - 2.00	117 (43%)	155 (57%)				38 (14%)	234 (86%)		
	MMP9_279	2.01 - 4.00	65 (41%)	95 (59%)				21 (13%)	139 (87%)		
	MMP9_279	>4.00	43 (36%)	78 (65%)	0.36	Trend		11 (9%)	110 (91%)	0.2	Trend
**Clark Level**	MMP9_279	I = in situ	27 (47%)	31 (53%)				9 (16%)	49 (85%)		
	MMP9_279	II	38 (35%)	70 (65%)				13 (12%)	95 (88%)		
	MMP9_279	III	64 (43%)	86 (57%)				21 (14%)	129 (86%)		
	MMP9_279	IV	194 (40%)	294 (60%)				67 (14%)	421 (86%)		
	MMP9_279	V	26 (38%)	43 (62%)	0.64	Trend		7 (10%)	62 (90%)	0.72	Trend
**Tumor Infiltrating Lymphocytes**	MMP9_279	absent	89 (43%)	117 (57%)				19 (9%)	187 (91%)		
	MMP9_279	non-brisk	155 (39%)	241 (61%)				46 (12%)	350 (88%)		
	MMP9_279	brisk	14 (45%)	17 (55%)	0.6	Trend		4 (13%)	27 (87%)	0.36	Trend
**Number of Moles**	MMP9_279	none	117 (45%)	143 (55%)				36 (14%)	224 (86%)		
	MMP9_279	few	201 (40%)	296 (60%)				64 (13%)	433 (87%)		
	MMP9_279	moderate	69 (41%)	100 (59%)				22 (13%)	147 (87%)		
	MMP9_279	many	17 (43%)	23 (58%)	0.44	Trend		5 (13%)	35 (88%)	0.76	Trend
**Number of Freckles**	MMP9_279	1 = none	198 (41%)	283 (59%)				61 (13%)	420 (87%)		
	MMP9_279	2	114 (48%)	126 (53%)				26 (11%)	214 (89%)		
	MMP9_279	3	70 (41%)	102 (59%)				25 (15%)	147 (86%)		
	MMP9_279	4	23 (35%)	42 (65%)				13 (20%)	52 (80%)		
	MMP9_279	5	6 (35%)	11 (65%)				1 (6%)	16 (94%)		
	MMP9_279	6 = many	3 (50%)	3 (50%)	0.64	Trend		0 (0%)	6 (100%)	0.56	Trend
**Phenotypic Index**	MMP9_279	1 = low risk	8 (21%)	30 (79%)				7 (18%)	31 (82%)		
	MMP9_279	2	99 (47%)	111 (53%)				27 (13%)	183 (87%)		
	MMP9_279	3	129 (41%)	187 (59%)				44 (14%)	272 (86%)		
	MMP9_279	4	145 (44%)	183 (56%)				34 (10%)	294 (90%)		
	MMP9_279	5 = high risk	39 (37%)	66 (63%)	0.92	Trend		17 (16%)	88 (84%)	0.6	Trend
**Sex**	MMP9_279	F	185 (43%)	244 (57%)				52 (12%)	377 (88%)		
	MMP9_279	M	235 (41%)	337 (59%)	0.52	Fisher		78 (14%)	494 (86%)	0.52	Fisher
**Family History**	MMP9_279	N	352 (43%)	473 (57%)				105 (13%)	720 (87%)		
	MMP9_279	Y	65 (39%)	102 (61%)	0.4	Fisher		24 (14%)	143 (86%)	0.6	Fisher
**Multiple Primary**	MMP9_279	N	361 (43%)	488 (58%)				109 (13%)	740 (87%)		
	MMP9_279	Y	59 (39%)	92 (61%)	0.48	Fisher		21 (14%)	130 (86%)	0.68	Fisher
**Dysplastic Nevus**	MMP9_279	N	239 (44%)	304 (56%)				69 (13%)	474 (87%)		
	MMP9_279	Y	80 (37%)	138 (63%)	0.08	Fisher		35 (16%)	183 (84%)	0.24	Fisher
**Ulceration**	MMP9_279	absent	213 (40%)	324 (60%)				70 (13%)	467 (87%)		
	MMP9_279	present	77 (41%)	113 (60%)	0.88	Fisher		24 (13%)	166 (87%)	1	Fisher
**Regression**	MMP9_279	absent	165 (44%)	212 (56%)				38 (10%)	339 (90%)		
	MMP9_279	present	100 (38%)	162 (62%)	0.16	Fisher		32 (12%)	230 (88%)	0.44	Fisher
**Lymphovascular Invasion**	MMP9_279	absent	230 (40%)	339 (60%)				68 (12%)	501 (88%)		
	MMP9_279	present	28 (41%)	40 (59%)	0.88	Fisher		7 (10%)	61 (90%)	0.84	Fisher
**Perineural Invasion**	MMP9_279	absent	174 (41%)	254 (59%)				52 (12%)	376 (88%)		
	MMP9_279	present	25 (40%)	37 (60%)	1	Fisher		5 (8%)	57 (92%)	0.52	Fisher
**Mitotic Index**	MMP9_279	absent	42 (41%)	61 (59%)				13 (13%)	90 (87%)		
	MMP9_279	present	188 (41%)	273 (59%)	1	Fisher		53 (12%)	408 (89%)	0.72	Fisher
**Satellites**	MMP9_279	absent	110 (43%)	144 (57%)				18 (7%)	236 (93%)		

	Polymorphism	variable	Homozygote Reference	Any Variant	p-value	Test		Homozygote Variant	Any Reference	p-value	Test
	MMP9_279	present	13 (43%)	17 (57%)	1	Fisher		4 (13%)	26 (87%)	0.28	Fisher
**Solar Elastosis**	MMP9_279	absent	15 (68%)	7 (32%)				0 (0%)	22 (100%)		
	MMP9_279	present	15 (38%)	25 (63%)	0.04	Fisher		4 (10%)	36 (90%)	0.28	Fisher
**Distant Metastasis**	MMP9_279	N	360 (41%)	509 (59%)				112 (13%)	757 (87%)		
	MMP9_279	Y	60 (46%)	70 (54%)	0.36	Fisher		18 (14%)	112 (86%)	0.76	Fisher
**Intransit Metastasis**	MMP9_279	N	384 (41%)	557 (59%)				122 (13%)	819 (87%)		
	MMP9_279	Y	17 (63%)	10 (37%)	0.04	Fisher		3 (11%)	24 (89%)	1	Fisher
**Tan/Burn Index**	MMP9_279	tend to tan	26 (29%)	65 (71%)				17 (19%)	74 (81%)		
	MMP9_279	tend to sunburn	394 (43%)	516 (57%)	<0.01	Fisher		113 (12%)	797 (88%)	0.12	Fisher
**Race**	MMP9_279	White non-Hispanic	401 (42%)	559 (58%)				127 (13%)	833 (87%)		
	MMP9_279	Hispanic	6 (55%)	5 (46%)				0 (0%)	11 (100%)		
	MMP9_279	Black non-Hispanic	5 (46%)	6 (55%)				1 (9%)	10 (91%)		
	MMP9_279	Asian/Indian	1 (33%)	2 (67%)	0.84	Chi-Square		0 (0%)	3 (100%)	0.52	Chi-Square
**Site**	MMP9_279	extremities	212 (40%)	323 (60%)				75 (14%)	460 (86%)		
	MMP9_279	head & neck	25 (35%)	46 (65%)				16 (23%)	55 (78%)		
	MMP9_279	non-cutaneous	6 (50%)	6 (50%)				2 (17%)	10 (83%)		
	MMP9_279	trunk	152 (45%)	189 (55%)	0.32	Chi-Square		34 (10%)	307 (90%)	0.04	Chi-Square
**Histology**	MMP9_279	desmoplastic	7 (30%)	16 (70%)				4 (17%)	19 (83%)		
	MMP9_279	other	91 (37%)	153 (63%)				31 (13%)	213 (87%)		
	MMP9_279	spitzoid	3 (30%)	7 (70%)				1 (10%)	9 (90%)		
	MMP9_279	unknown	319 (44%)	405 (56%)	0.16	Chi-Square		94 (13%)	630 (87%)	0.92	Chi-Square
**Stage at Diagnosis**	MMP9_574	0	28 (90%)	3 (10%)				1 (3%)	30 (97%)		
	MMP9_574	I	257 (92%)	21 (8%)				0 (0%)	278 (100%)		
	MMP9_574	II	100 (89%)	13 (12%)				0 (0%)	113 (100%)		
	MMP9_574	III	84 (89%)	10 (11%)				0 (0%)	94 (100%)		
	MMP9_574	IV	3 (75%)	1 (25%)	0.24	Trend		0 (0%)	4 (100%)	0.08	Trend
**Current Stage**	MMP9_574	0	27 (90%)	3 (10%)				1 (3%)	29 (97%)		
	MMP9_574	I	221 (92%)	19 (8%)				0 (0%)	240 (100%)		
	MMP9_574	II	68 (88%)	9 (12%)				0 (0%)	77 (100%)		
	MMP9_574	III	87 (90%)	10 (10%)				0 (0%)	97 (100%)		
	MMP9_574	IV	79 (92%)	7 (8%)	0.84	Trend		0 (0%)	86 (100%)	0.12	Trend
**Thickness**	MMP9_574	in situ	28 (90%)	3 (10%)				1 (3%)	30 (97%)		
	MMP9_574	<1.01	172 (93%)	14 (8%)				0 (0%)	186 (100%)		
	MMP9_574	1.01 - 2.00	126 (93%)	10 (7%)				0 (0%)	136 (100%)		
	MMP9_574	2.01 - 4.00	68 (85%)	12 (15%)				0 (0%)	80 (100%)		
	MMP9_574	>4.00	59 (92%)	5 (8%)	0.44	Trend		0 (0%)	64 (100%)	0.08	Trend
**Clark Level**	MMP9_574	I = in situ	28 (90%)	3 (10%)				1 (3%)	30 (97%)		
	MMP9_574	II	62 (93%)	5 (8%)				0 (0%)	67 (100%)		
	MMP9_574	III	79 (93%)	6 (7%)				0 (0%)	85 (100%)		
	MMP9_574	IV	217 (90%)	24 (10%)				0 (0%)	241 (100%)		
	MMP9_574	V	34 (87%)	5 (13%)	0.4	Trend		0 (0%)	39 (100%)	0.04	Trend
**Tumor Infiltrating Lymphocytes**	MMP9_574	absent	106 (89%)	13 (11%)				0 (0%)	119 (100%)		
	MMP9_574	non-brisk	162 (90%)	18 (10%)				0 (0%)	180 (100%)		
	MMP9_574	brisk	15 (94%)	1 (6%)	0.6	Trend		0 (0%)	16 (100%)	0.6	Trend
**Number of Moles**	MMP9_574	none	153 (92%)	14 (8%)				1 (1%)	166 (99%)		
	MMP9_574	few	211 (91%)	22 (9%)				0 (0%)	233 (100%)		

	Polymorphism	variable	Homozygote Reference	Any Variant	p-value	Test		Homozygote Variant	Any Reference	p-value	Test
	MMP9_574	moderate	72 (95%)	4 (5%)				0 (0%)	76 (100%)		
	MMP9_574	many	24 (86%)	4 (14%)	0.84	Trend		0 (0%)	28 (100%)	0.28	Trend
**Number of Freckles**	MMP9_574	1 = none	227 (90%)	25 (10%)				1 (0%)	251 (100%)		
	MMP9_574	2	124 (89%)	15 (11%)				0 (0%)	139 (100%)		
	MMP9_574	3	81 (93%)	6 (7%)				0 (0%)	87 (100%)		
	MMP9_574	4	32 (100%)	0 (0%)				0 (0%)	32 (100%)		
	MMP9_574	5	6 (100%)	0 (0%)				0 (0%)	6 (100%)		
	MMP9_574	6 = many	1 (50%)	1 (50%)	0.24	Trend		0 (0%)	2 (100%)	0.4	Trend
**Phenotypic Index**	MMP9_574	1 = low risk	15 (75%)	5 (25%)				1 (5%)	19 (95%)		
	MMP9_574	2	87 (88%)	12 (12%)				0 (0%)	99 (100%)		
	MMP9_574	3	165 (95%)	8 (5%)				0 (0%)	173 (100%)		
	MMP9_574	4	161 (90%)	17 (10%)				0 (0%)	178 (100%)		
	MMP9_574	5 = high risk	56 (90%)	6 (10%)	0.24	Trend		0 (0%)	62 (100%)	0.04	Trend
**Sex**	MMP9_574	F	220 (91%)	22 (9%)				1 (0%)	241 (100%)		
	MMP9_574	M	265 (91%)	26 (9%)	1	Fisher		0 (0%)	291 (100%)	0.44	Fisher
**Family History**	MMP9_574	N	395 (90%)	45 (10%)				0 (0%)	440 (100%)		
	MMP9_574	Y	88 (97%)	3 (3%)	0.04	Fisher		1 (1%)	90 (99%)	0.16	Fisher
**Multiple Primary**	MMP9_574	N	410 (90%)	45 (10%)				1 (0%)	454 (100%)		
	MMP9_574	Y	75 (96%)	3 (4%)	0.08	Fisher		0 (0%)	78 (100%)	1	Fisher
**Dysplastic Nevus**	MMP9_574	N	307 (90%)	33 (10%)				1 (0%)	339 (100%)		
	MMP9_574	Y	117 (93%)	9 (7%)	0.48	Fisher		0 (0%)	126 (100%)	1	Fisher
**Ulceration**	MMP9_574	absent	254 (91%)	25 (9%)				0 (0%)	279 (100%)		
	MMP9_574	present	85 (89%)	11 (12%)	0.56	Fisher		0 (0%)	96 (100%)	0.56	Fisher
**Regression**	MMP9_574	absent	181 (90%)	20 (10%)				0 (0%)	201 (100%)		
	MMP9_574	present	104 (90%)	12 (10%)	1	Fisher		0 (0%)	116 (100%)	1	Fisher
**Lymphovascular Invasion**	MMP9_574	absent	267 (90%)	30 (10%)				0 (0%)	297 (100%)		
	MMP9_574	present	26 (93%)	2 (7%)	1	Fisher		0 (0%)	28 (100%)	1	Fisher
**Perineural Invasion**	MMP9_574	absent	176 (90%)	20 (10%)				0 (0%)	196 (100%)		
	MMP9_574	present	26 (90%)	3 (10%)	1	Fisher		0 (0%)	29 (100%)	1	Fisher
**Mitotic Index**	MMP9_574	absent	58 (92%)	5 (8%)				0 (0%)	63 (100%)		
	MMP9_574	present	184 (89%)	23 (11%)	0.64	Fisher		0 (0%)	207 (100%)	0.64	Fisher
**Satellites**	MMP9_574	absent	116 (89%)	14 (11%)				0 (0%)	130 (100%)		
	MMP9_574	present	14 (93%)	1 (7%)	1	Fisher		0 (0%)	15 (100%)	1	Fisher
**Solar Elastosis**	MMP9_574	absent	11 (92%)	1 (8%)				0 (0%)	12 (100%)		
	MMP9_574	present	22 (85%)	4 (15%)	1	Fisher		0 (0%)	26 (100%)	1	Fisher
**Distant Metastasis**	MMP9_574	N	405 (91%)	41 (9%)				1 (0%)	445 (100%)		
	MMP9_574	Y	79 (92%)	7 (8%)	1	Fisher		0 (0%)	86 (100%)	1	Fisher
**Intransit Metastasis**	MMP9_574	N	449 (92%)	40 (8%)				1 (0%)	488 (100%)		
	MMP9_574	Y	19 (91%)	2 (10%)	0.68	Fisher		0 (0%)	21 (100%)	1	Fisher
**Tan/Burn Index**	MMP9_574	tend to tan	45 (90%)	5 (10%)				1 (2%)	49 (98%)		
	MMP9_574	tend to sunburn	440 (91%)	43 (9%)	0.8	Fisher		0 (0%)	483 (100%)	0.08	Fisher
**Race**	MMP9_574	White non-Hispanic	465 (92%)	43 (9%)				1 (0%)	507 (100%)		
	MMP9_574	Hispanic	5 (63%)	3 (38%)				0 (0%)	8 (100%)		
	MMP9_574	Black non-Hispanic	4 (67%)	2 (33%)				0 (0%)	6 (100%)		
	MMP9_574	Asian/Indian	2 (100%)	0 (0%)	<0.01	Chi-Square		0 (0%)	2 (100%)	1	Chi-Square
**Site**	MMP9_574	extremities	263 (88%)	36 (12%)				0 (0%)	299 (100%)		

	Polymorphism	variable	Homozygote Reference	Any Variant	p-value	Test		Homozygote Variant	Any Reference	p-value	Test
	MMP9_574	head & neck	36 (97%)	1 (3%)				0 (0%)	37 (100%)		
	MMP9_574	non-cutaneous	6 (100%)	0 (0%)				0 (0%)	6 (100%)		
	MMP9_574	trunk	164 (96%)	7 (4%)	<0.01	Chi-Square		1 (1%)	170 (99%)	0.56	Chi-Square
**Histology**	MMP9_574	desmoplastic	7 (88%)	1 (13%)				0 (0%)	8 (100%)		
	MMP9_574	other	117 (91%)	12 (9%)				0 (0%)	129 (100%)		
	MMP9_574	spitzoid	2 (50%)	2 (50%)				0 (0%)	4 (100%)		
	MMP9_574	unknown	359 (92%)	33 (8%)	0.04	Chi-Square		1 (0%)	391 (100%)	0.96	Chi-Square
**Stage at Diagnosis**	MMP9_668	0	38 (66%)	20 (35%)				2 (3%)	56 (97%)		
	MMP9_668	I	355 (71%)	147 (29%)				12 (2%)	490 (98%)		
	MMP9_668	II	164 (70%)	69 (30%)				1 (0%)	232 (100%)		
	MMP9_668	III	130 (77%)	38 (23%)				3 (2%)	165 (98%)		
	MMP9_668	IV	8 (89%)	1 (11%)	0.04	Trend		0 (0%)	9 (100%)	0.16	Trend
**Current Stage**	MMP9_668	0	36 (64%)	20 (36%)				2 (4%)	54 (96%)		
	MMP9_668	I	302 (70%)	131 (30%)				11 (3%)	422 (98%)		
	MMP9_668	II	114 (72%)	44 (28%)				0 (0%)	158 (100%)		
	MMP9_668	III	163 (76%)	53 (25%)				4 (2%)	212 (98%)		
	MMP9_668	IV	98 (77%)	30 (23%)	0.04	Trend		2 (2%)	126 (98%)	0.28	Trend
**Thickness**	MMP9_668	in situ	38 (66%)	20 (35%)				2 (3%)	56 (97%)		
	MMP9_668	<1.01	219 (69%)	100 (31%)				9 (3%)	310 (97%)		
	MMP9_668	1.01 - 2.00	205 (75%)	67 (25%)				3 (1%)	269 (99%)		
	MMP9_668	2.01 - 4.00	115 (72%)	44 (28%)				3 (2%)	156 (98%)		
	MMP9_668	>4.00	84 (70%)	36 (30%)	0.4	Trend		1 (1%)	119 (99%)	0.12	Trend
**Clark Level**	MMP9_668	I = in situ	38 (66%)	20 (35%)				2 (3%)	56 (97%)		
	MMP9_668	II	73 (68%)	34 (32%)				2 (2%)	105 (98%)		
	MMP9_668	III	101 (67%)	49 (33%)				7 (5%)	143 (95%)		
	MMP9_668	IV	355 (73%)	130 (27%)				5 (1%)	480 (99%)		
	MMP9_668	V	45 (65%)	24 (35%)	0.32	Trend		1 (1%)	68 (99%)	0.12	Trend
**Tumor Infiltrating Lymphocytes**	MMP9_668	absent	146 (71%)	59 (29%)				4 (2%)	201 (98%)		
	MMP9_668	non-brisk	279 (71%)	114 (29%)				5 (1%)	388 (99%)		
	MMP9_668	brisk	24 (77%)	7 (23%)	0.72	Trend		0 (0%)	31 (100%)	0.36	Trend
**Number of Moles**	MMP9_668	none	183 (71%)	76 (29%)				8 (3%)	251 (97%)		
	MMP9_668	few	359 (72%)	137 (28%)				9 (2%)	487 (98%)		
	MMP9_668	moderate	119 (71%)	48 (29%)				1 (1%)	166 (99%)		
	MMP9_668	many	30 (75%)	10 (25%)	0.68	Trend		1 (3%)	39 (98%)	0.16	Trend
**Number of Freckles**	MMP9_668	1 = none	338 (71%)	140 (29%)				6 (1%)	472 (99%)		
	MMP9_668	2	183 (76%)	57 (24%)				3 (1%)	237 (99%)		
	MMP9_668	3	122 (71%)	49 (29%)				5 (3%)	166 (97%)		
	MMP9_668	4	44 (68%)	21 (32%)				4 (6%)	61 (94%)		
	MMP9_668	5	12 (71%)	5 (29%)				0 (0%)	17 (100%)		
	MMP9_668	6 = many	4 (67%)	2 (33%)	0.88	Trend		0 (0%)	6 (100%)	0.04	Trend
**Phenotypic Index**	MMP9_668	1 = low risk	20 (53%)	18 (47%)				0 (0%)	38 (100%)		
	MMP9_668	2	160 (77%)	48 (23%)				2 (1%)	206 (99%)		
	MMP9_668	3	222 (71%)	93 (30%)				10 (3%)	305 (97%)		
	MMP9_668	4	240 (73%)	87 (27%)				5 (2%)	322 (99%)		
	MMP9_668	5 = high risk	73 (70%)	32 (31%)	0.8	Trend		2 (2%)	103 (98%)	0.64	Trend
**Sex**	MMP9_668	F	311 (73%)	116 (27%)				7 (2%)	420 (98%)		

	Polymorphism	variable	Homozygote Reference	Any Variant	p-value	Test		Homozygote Variant	Any Reference	p-value	Test
	MMP9_668	M	405 (71%)	165 (29%)	0.56	Fisher		12 (2%)	558 (98%)	0.64	Fisher
**Family History**	MMP9_668	N	598 (73%)	225 (27%)				14 (2%)	809 (98%)		
	MMP9_668	Y	113 (69%)	52 (32%)	0.28	Fisher		5 (3%)	160 (97%)	0.36	Fisher
**Multiple Primary**	MMP9_668	N	612 (72%)	235 (28%)				17 (2%)	830 (98%)		
	MMP9_668	Y	104 (70%)	45 (30%)	0.56	Fisher		2 (1%)	147 (99%)	0.76	Fisher
**Dysplastic Nevus**	MMP9_668	N	395 (73%)	148 (27%)				11 (2%)	532 (98%)		
	MMP9_668	Y	145 (67%)	72 (33%)	0.12	Fisher		4 (2%)	213 (98%)	1	Fisher
**Ulceration**	MMP9_668	absent	379 (71%)	156 (29%)				12 (2%)	523 (98%)		
	MMP9_668	present	135 (72%)	53 (28%)	0.84	Fisher		2 (1%)	186 (99%)	0.52	Fisher
**Regression**	MMP9_668	absent	271 (72%)	105 (28%)				4 (1%)	372 (99%)		
	MMP9_668	present	180 (70%)	79 (31%)	0.52	Fisher		5 (2%)	254 (98%)	0.48	Fisher
**Lymphovascular Invasion**	MMP9_668	absent	400 (71%)	165 (29%)				9 (2%)	556 (98%)		
	MMP9_668	present	50 (74%)	18 (27%)	0.68	Fisher		2 (3%)	66 (97%)	0.32	Fisher
**Perineural Invasion**	MMP9_668	absent	303 (72%)	121 (29%)				7 (2%)	417 (98%)		
	MMP9_668	present	42 (68%)	20 (32%)	0.56	Fisher		0 (0%)	62 (100%)	0.6	Fisher
**Mitotic Index**	MMP9_668	absent	73 (72%)	29 (28%)				2 (2%)	100 (98%)		
	MMP9_668	present	324 (71%)	134 (29%)	0.92	Fisher		6 (1%)	452 (99%)	0.64	Fisher
**Satellites**	MMP9_668	absent	189 (75%)	63 (25%)				1 (0%)	251 (100%)		
	MMP9_668	present	25 (83%)	5 (17%)	0.36	Fisher		0 (0%)	30 (100%)	1	Fisher
**Solar Elastosis**	MMP9_668	absent	18 (82%)	4 (18%)				0 (0%)	22 (100%)		
	MMP9_668	present	30 (75%)	10 (25%)	0.76	Fisher		0 (0%)	40 (100%)	0.76	Fisher
**Distant Metastasis**	MMP9_668	N	617 (71%)	249 (29%)				17 (2%)	849 (98%)		
	MMP9_668	Y	99 (77%)	30 (23%)	0.2	Fisher		2 (2%)	127 (98%)	1	Fisher
**Intransit Metastasis**	MMP9_668	N	667 (71%)	270 (29%)				18 (2%)	919 (98%)		
	MMP9_668	Y	22 (82%)	5 (19%)	0.28	Fisher		1 (4%)	26 (96%)	0.44	Fisher
**Tan/Burn Index**	MMP9_668	tend to tan	55 (60%)	36 (40%)				2 (2%)	89 (98%)		
	MMP9_668	tend to sunburn	661 (73%)	245 (27%)	<0.01	Fisher		17 (2%)	889 (98%)	0.68	Fisher
**Race**	MMP9_668	White non-Hispanic	687 (72%)	269 (28%)				18 (2%)	938 (98%)		
	MMP9_668	Hispanic	7 (64%)	4 (36%)				0 (0%)	11 (100%)		
	MMP9_668	Black non-Hispanic	7 (64%)	4 (36%)				1 (9%)	10 (91%)		
	MMP9_668	Asian/Indian	2 (67%)	1 (33%)	0.88	Chi-Square		0 (0%)	3 (100%)	0.36	Chi-Square
**Site**	MMP9_668	extremities	375 (70%)	158 (30%)				12 (2%)	521 (98%)		
	MMP9_668	head & neck	49 (69%)	22 (31%)				1 (1%)	70 (99%)		
	MMP9_668	non-cutaneous	6 (55%)	5 (46%)				2 (18%)	9 (82%)		
	MMP9_668	trunk	252 (74%)	88 (26%)	0.36	Chi-Square		4 (1%)	336 (99%)	<0.01	Chi-Square
**Histology**	MMP9_668	desmoplastic	16 (70%)	7 (30%)				0 (0%)	23 (100%)		
	MMP9_668	other	170 (70%)	72 (30%)				4 (2%)	238 (98%)		
	MMP9_668	spitzoid	8 (80%)	2 (20%)				0 (0%)	10 (100%)		
	MMP9_668	unknown	522 (72%)	200 (28%)	0.84	Chi-Square		15 (2%)	707 (98%)	0.84	Chi-Square
