# Supplementary material for: Matrix Metalloproteinase-9 (MMP-9) polymorphisms in patients with cutaneous malignant melanoma
Source: BMC Med Genet. 2007 Mar 8;8:10. doi: 10.1186/1471-2350-8-10 (PMC1831467; doi:10.1186/1471-2350-8-10)
Supplement: Additional file 4 — Genotyping and statistical analysis for microsatellite. The table shows the statistical analysis of the genotype frequencies for the microsatellite and all variables studied [file 1471-2350-8-10-S4.pdf]

Supplementary Table 4: Genotyping and statistical results for microsatellite

	Polymorphism	variable	At least one $[CA]_{14}$	Absence of $[CA]_{14}$	p-value	Test	At least one $[CA]_{\geq 21}$	Absence of $[CA]_{\geq 21}$	p-value	Test
**Stage at Diagnosis**	microsatellite	0	42 (74%)	15 (26%)			40 (70%)	17 (30%)		
	microsatellite	I	383 (79%)	101 (21%)			321 (66%)	163 (34%)		
	microsatellite	II	175 (77%)	52 (23%)			146 (64%)	81 (36%)		
	microsatellite	III	129 (80%)	32 (20%)			100 (62%)	61 (38%)		
	microsatellite	IV	7 (78%)	2 (22%)	0,68	Trend	7 (78%)	2 (22%)	0,32	Trend
**Current Stage**	microsatellite	0	41 (75%)	14 (26%)			39 (71%)	16 (29%)		
	microsatellite	I	329 (79%)	87 (21%)			274 (66%)	142 (34%)		
	microsatellite	II	117 (77%)	36 (24%)			95 (62%)	58 (38%)		
	microsatellite	III	163 (78%)	46 (22%)			135 (65%)	74 (35%)		
	microsatellite	IV	101 (81%)	24 (19%)	0,68	Trend	82 (66%)	43 (34%)	0,6	Trend
**Thickness**	microsatellite	in situ	42 (74%)	15 (26%)			40 (70%)	17 (30%)		
	microsatellite	<1.01	244 (79%)	64 (21%)			203 (66%)	105 (34%)		
	microsatellite	1.01 - 2.00	206 (79%)	56 (21%)			171 (65%)	91 (35%)		
	microsatellite	2.01 - 4.00	116 (76%)	36 (24%)			102 (67%)	50 (33%)		
	microsatellite	>4.00	93 (80%)	24 (21%)	0,88	Trend	76 (65%)	41 (35%)	0,76	Trend
**Clark Level**	microsatellite	I = in situ	42 (74%)	15 (26%)			40 (70%)	17 (30%)		
	microsatellite	II	83 (79%)	22 (21%)			70 (67%)	35 (33%)		
	microsatellite	III	115 (79%)	31 (21%)			91 (62%)	55 (38%)		
	microsatellite	IV	366 (79%)	97 (21%)			312 (67%)	151 (33%)		
	microsatellite	V	47 (70%)	20 (30%)	0,92	Trend	44 (66%)	23 (34%)	0,92	Trend
**Tumor Infiltrating Lymphocytes**	microsatellite	absent	166 (85%)	30 (15%)			129 (66%)	67 (34%)		
	microsatellite	non-brisk	300 (79%)	82 (22%)			251 (66%)	131 (34%)		
	microsatellite	brisk	23 (77%)	7 (23%)	0,08	Trend	21 (70%)	9 (30%)	0,8	Trend
**Number of Moles**	microsatellite	none	193 (79%)	52 (21%)			156 (64%)	89 (36%)		
	microsatellite	few	379 (78%)	107 (22%)			318 (65%)	168 (35%)		
	microsatellite	moderate	126 (78%)	35 (22%)			109 (68%)	52 (32%)		
	microsatellite	many	30 (77%)	9 (23%)	0,8	Trend	26 (67%)	13 (33%)	0,44	Trend
**Number of Freckles**	microsatellite	1 = none	358 (78%)	100 (22%)			309 (68%)	149 (33%)		
	microsatellite	2	183 (79%)	49 (21%)			137 (59%)	95 (41%)		
	microsatellite	3	129 (77%)	38 (23%)			111 (67%)	56 (34%)		
	microsatellite	4	50 (78%)	14 (22%)			40 (63%)	24 (38%)		
	microsatellite	5	14 (82%)	3 (18%)			12 (71%)	5 (29%)		
	microsatellite	6 = many	6 (100%)	0 (0%)	0,68	Trend	5 (83%)	1 (17%)	0,76	Trend
**Phenotypic Index**	microsatellite	1 = low risk	19 (54%)	16 (46%)			31 (89%)	4 (11%)		
	microsatellite	2	158 (78%)	46 (23%)			139 (68%)	65 (32%)		
	microsatellite	3	237 (79%)	65 (22%)			190 (63%)	112 (37%)		
	microsatellite	4	260 (82%)	56 (18%)			197 (62%)	119 (38%)		
	microsatellite	5 = high risk	79 (77%)	24 (23%)	0,04	Trend	69 (67%)	34 (33%)	0,08	Trend
**Sex**	microsatellite	F	327 (80%)	82 (20%)			260 (64%)	149 (36%)		
	microsatellite	M	429 (77%)	126 (23%)	0,36	Fisher	370 (67%)	185 (33%)	0,32	Fisher
**Family History**	microsatellite	N	630 (79%)	171 (21%)			523 (65%)	278 (35%)		
	microsatellite	Y	119 (77%)	36 (23%)	0,6	Fisher	102 (66%)	53 (34%)	0,92	Fisher
**Multiple Primary**	microsatellite	N	641 (78%)	178 (22%)			533 (65%)	286 (35%)		
	microsatellite	Y	114 (79%)	30 (21%)	0,92	Fisher	96 (67%)	48 (33%)	0,76	Fisher

	Polymorphism	variable	At least one $[CA]_{14}$	Absence of $[CA]_{14}$	p-value	Test	At least one $[CA]_{\geq 21}$	Absence of $[CA]_{\geq 21}$	p-value	Test
**Dysplastic Nevus**	microsatellite	N	414 (80%)	106 (20%)			336 (65%)	184 (35%)		
	microsatellite	Y	159 (76%)	51 (24%)	0,28	Fisher	142 (68%)	68 (32%)	0,48	Fisher
**Ulceration**	microsatellite	absent	403 (78%)	112 (22%)			340 (66%)	175 (34%)		
	microsatellite	present	142 (78%)	40 (22%)	1	Fisher	120 (66%)	62 (34%)	1	Fisher
**Regression**	microsatellite	absent	298 (82%)	64 (18%)			228 (63%)	134 (37%)		
	microsatellite	present	190 (76%)	59 (24%)	0,08	Fisher	171 (69%)	78 (31%)	0,16	Fisher
**Lymphovascular Invasion**	microsatellite	absent	436 (80%)	111 (20%)			362 (66%)	185 (34%)		
	microsatellite	present	50 (77%)	15 (23%)	0,64	Fisher	43 (66%)	22 (34%)	1	Fisher
**Perineural Invasion**	microsatellite	absent	318 (78%)	89 (22%)			267 (66%)	140 (34%)		
	microsatellite	present	49 (82%)	11 (18%)	0,6	Fisher	39 (65%)	21 (35%)	1	Fisher
**Mitotic Index**	microsatellite	absent	77 (79%)	20 (21%)			70 (72%)	27 (28%)		
	microsatellite	present	351 (80%)	89 (20%)	1	Fisher	287 (65%)	153 (35%)	0,24	Fisher
**Satellites**	microsatellite	absent	206 (84%)	39 (16%)			160 (65%)	85 (35%)		
	microsatellite	present	24 (80%)	6 (20%)	0,6	Fisher	17 (57%)	13 (43%)	0,4	Fisher
**Solar Elastosis**	microsatellite	absent	18 (95%)	1 (5%)			9 (47%)	10 (53%)		
	microsatellite	present	29 (76%)	9 (24%)	0,16	Fisher	26 (68%)	12 (32%)	0,16	Fisher
**Distant Metastasis**	microsatellite	N	652 (78%)	184 (22%)			546 (65%)	290 (35%)		
	microsatellite	Y	102 (81%)	24 (19%)	0,48	Fisher	82 (65%)	44 (35%)	1	Fisher
**Intransit Metastasis**	microsatellite	N	710 (78%)	196 (22%)			598 (66%)	308 (34%)		
	microsatellite	Y	20 (77%)	6 (23%)	0,8	Fisher	12 (46%)	14 (54%)	0,04	Fisher
**Tan/Burn Index**	microsatellite	tend to tan	57 (66%)	30 (35%)			68 (78%)	19 (22%)		
	microsatellite	tend to sunburn	699 (80%)	178 (20%)	<0.01	Fisher	562 (64%)	315 (36%)	<0.01	Fisher
**Race**	microsatellite	White non-Hispanic	733 (79%)	194 (21%)			601 (65%)	326 (35%)		
	microsatellite	Hispanic	7 (70%)	3 (30%)			8 (80%)	2 (20%)		
	microsatellite	Black non-Hispanic	3 (33%)	6 (67%)			8 (89%)	1 (11%)		
	microsatellite	Asian/Indian	1 (33%)	2 (67%)	<0.01	Chi-Square	3 (100%)	0 (0%)	0,2	Chi-Square
**Site**	microsatellite	extremities	387 (75%)	130 (25%)			348 (67%)	169 (33%)		
	microsatellite	head & neck	51 (75%)	17 (25%)			44 (65%)	24 (35%)		
	microsatellite	non-cutaneous	9 (82%)	2 (18%)			6 (55%)	5 (46%)		
	microsatellite	trunk	274 (84%)	52 (16%)	<0.01	Chi-Square	209 (64%)	117 (36%)	0,68	Chi-Square
**Histology**	microsatellite	desmoplastic	16 (73%)	6 (27%)			14 (64%)	8 (36%)		
	microsatellite	other	179 (76%)	56 (24%)			165 (70%)	70 (30%)		
	microsatellite	spitzoid	8 (80%)	2 (20%)			8 (80%)	2 (20%)		
	microsatellite	unknown	553 (79%)	144 (21%)	0,68	Chi-Square	443 (64%)	254 (36%)	0,2	Chi-Square
